# Photoswitching of halide-binding affinity and selectivity in dithienylethene-strapped calix[4]pyrrole†

**DOI:** 10.1039/d3cc02264a

**Published:** 2023-06-22

**Authors:** David Villarón, Guido E. A. Brugman, Maxime A. Siegler, Sander J. Wezenberg

**Affiliations:** a Leiden Institute of Chemistry, Leiden University Einsteinweg 55 Leiden 2333 CC The Netherlands s.j.wezenberg@lic.leidenuniv.nl; b Department of Chemistry, Johns Hopkins University 3400 N, Charles St. Baltimore MD 21218 USA

## Abstract

Dithienylethene-strapped calix[4]pyrrole is isomerized by 300/630 nm light between ring-open and -closed isomers, which affects the size of the anion binding site. Where for chloride this results in only a small change in affinity, that of the larger bromide and iodide ions is majorly affected, resulting in altered selectivity.

## Abstract 2

Photoisomerization between ring-open and -closed dithienylethene-strapped calix[4]pyrrole alters binding affinity and selectivity towards halide anions.

## Graphical abstract

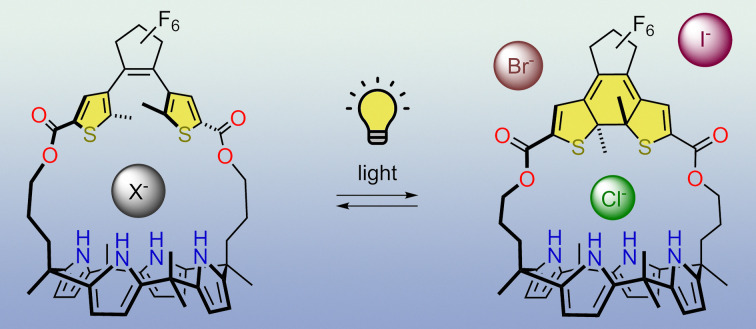

There is tremendous interest in the development of artificial anion receptors for their sensing,^1^ extraction,^2^ and transport properties.^3^ These properties could potentially be improved by achieving stimulus control of binding affinity.^4,5^ For example, it would enable recovery of receptor and substrate in sensing and extraction applications and allow (de)activation of membrane transport processes.^6^ Significant effort has therefore been put into conferring stimuli-responsive properties to anion receptors, with strategies that are based on light being particularly successful.^4,5^ It remains challenging, however, to achieve large differences in affinity between photoaddressable states. Moreover, while switching of the ion selectivity of protein channels is known to have important implications in cell physiology,^7^ selectivity control in photoresponsive anion receptors has only been reported for bis-carboxylate^5*f*,*j*^ and bis-sulfonate guests^5*m*^ with different spacer length, and for the enantiomers of BINOL-derived phosphate.^5*g*^

Among synthetic anion receptors, calix[4]pyrrole is privileged for its strong binding towards halide ions, in particular chloride.^8^ Importantly, the groups of Lee and Sessler described that their binding strength can be enhanced by bridging the distal *meso*-positions in so named strapped calix[4]pyrroles.^9^ Variations in the nature and length of the strapping unit were found to influence binding affinity, as well as Cl^−^/Br^−^ selectivity, presumably because of changes in size complementarity between the anion and the binding cavity that is created by the strap.^10^ Recently, we introduced a stiff-stilbene photoswitch into the bridging unit, which gave rise to an 8000-fold difference in affinity for chloride between (*E*)- and (*Z*)-isomer.^5*k*^ Stiff-stilbene is structurally rigid and undergoes a large geometrical change upon isomerization, which is deemed crucial in attaining such a substantial change in binding affinity.^11^ Nevertheless, we envisioned that a more subtle structural change could alter halide-size selectivity, in addition to affinity. Hence, we turned our attention to dithienylethene (DTE), which is a derivative of stilbene and isomerizes between a ring-open and -closed form.^12^ This photo-chromic switch has been widely applied in (opto-electronic) materials and is known for its efficient isomerization and high fatigue resistance. Its application in photoresponsive host–guest systems, on the other hand, is fairly limited,^5*b*,13^ and generally results in moderate affinity differences, except for the Pd_2_L_4_ metal–organic cages comprised of DTE ligands that have been reported by the group of Clever.^5*c*,*m*^

Herein, DTE-strapped calix[4]pyrrole receptor 1 is presented (Scheme 1). Photoisomerization between open (*o*) and closed (*c*) isomers comes with a change in the size of the binding site. While the decrease in affinity for Cl^−^ upon ring-closing is small (2.2-fold), it is reduced by about 160 times for the larger Br^−^ ion whereas for I^−^ the binding becomes too weak to be quantified. The selectivity towards Cl^−^ is thus greatly enhanced. Further, the change in Br^−^ binding affinity is – to our knowledge – the largest achieved so far in photoswitchable receptors.^4,5^

**Scheme 1:**
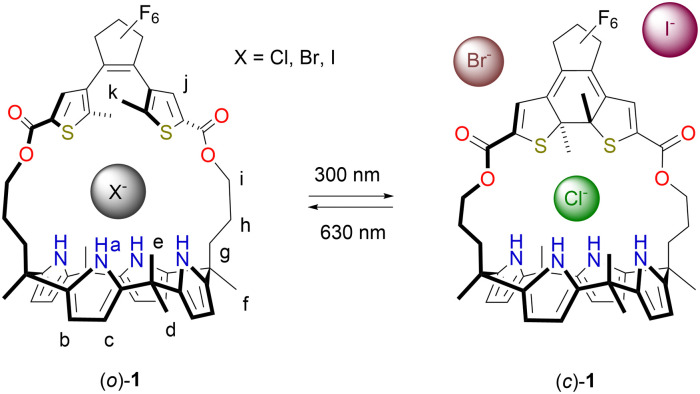
Photoswitching of halide binding affinity and size selectivity.

The synthesis of (*o*)-1 is outlined in Scheme 2 and started with previously reported dichloro-substituted DTE (*o*)-2.^14^ Lithiation of this compound followed by carboxylation, using a procedure adapted from the literature,^15^ afforded dicarboxylic acid (*o*)-3. Subsequent esterification with known dipyrromethane alcohol 5,^10^ which was mediated by (benzotriazol-1-yloxy)dipiperidino-carbenium tetrafluoroborate (TBTU), gave bis-dipyrromethane (*o*)-4. This bis-dipyrromethane precursor was then condensed with acetone in the presence of a catalytic amount of boron trifluoride to obtain the desired strapped calix[4]pyrrole receptor (*o*)-1 (see the ESI† for details and characterization).

**Scheme 2:**
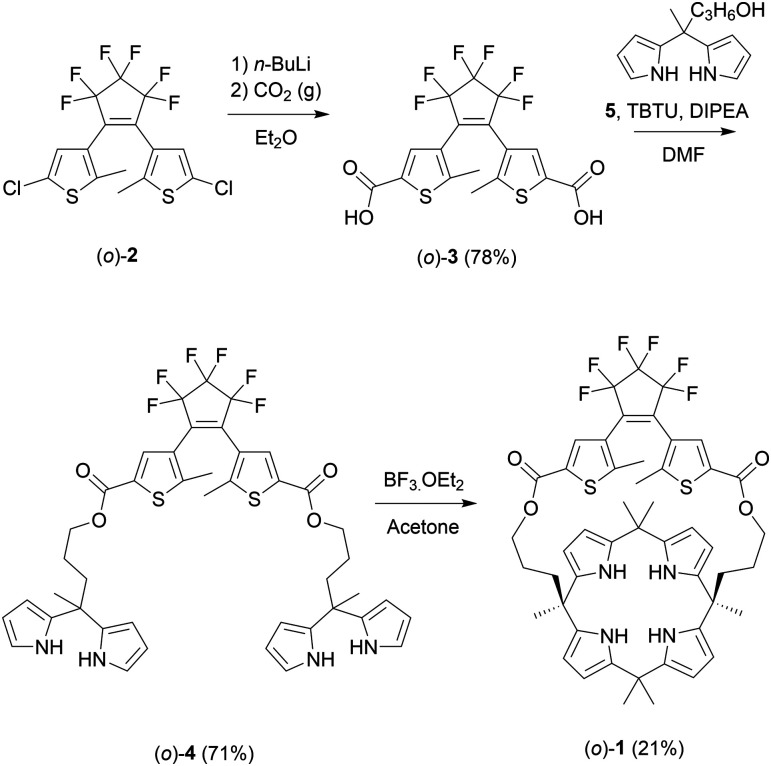
Synthesis of dithienylethene-strapped calix[4]pyrrole receptor (*o*)-1.

The photoswitching properties were first studied by UV-Vis spectroscopy in MeCN solution. Compound (*o*)-1 displayed an absorption maximum at *λ* = 227 nm (Fig. 1A). Irradiation with 300 nm light led to the appearance of two new absorption maxima at *λ* = 379 nm and at 600 nm, while the absorption between 230–300 nm decreased. These light-induced spectral changes are similar to what has been reported for other DTE-based systems and can be ascribed to formation of the ring-closed isomer.^12^ When the same sample was subsequently irradiated with 630 nm light, an absorption spectrum closely identical to the original one of (*o*)-1 was obtained, illustrating photoinduced ring-opening. At both wavelengths irradiation was stopped when no further changes in absorption were noted, *i.e.*, when the photostationary state (PSS) had been reached. The conservation of isosbestic points at *λ* = 231 and 305 nm pointed to a unimolecular process and furthermore, multiple 300/630 nm alternating cycles showed only minimal signs of fatigue (Fig. S10A–C in the ESI†).

**Fig. 1:**
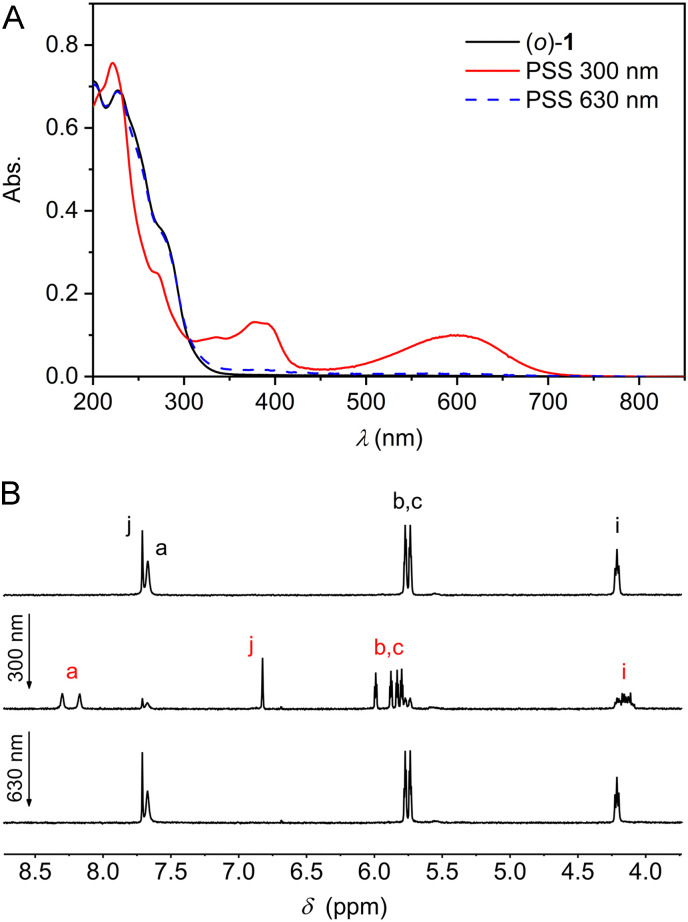
(A) UV-Vis (2.1 × 10^−5^ m in MeCN) and (B) ^1^H NMR (2.0 × 10^−3^ m in MeCN-*d*_3_) spectral changes starting with (*o*)-1 upon consecutive 300 nm and 630 nm irradiation. For the lettering assignment of protons, see Scheme 1.

Next, the photoisomerization processes was monitored by ^1^H NMR spectroscopy. Irradiation of a MeCN-*d*_3_ solution of (*o*)-1 with 300 nm light led to the appearance of a new set of ^1^H NMR signals, which could be assigned to the ring-closed isomer (Fig. 1B and Fig. S11 in the ESI†). Most notably, the aromatic singlet for the thiophene protons (H_j_) moved upfield (from *δ* = 7.71 to 6.82 ppm), whereas the singlet for the pyrrole NH protons (H_a_) and the two double doublets for the β-pyrrolic protons (H_b_ and H_c_) splitted into downfield-shifted signals (from *δ* = 7.67 into 8.30 and 8.18 ppm and from *δ* = 5.77 and 5.74 into 5.99, 5.88, 5.83, and 5.80 ppm, respectively). By signal integration, the PSS_300_ (*o*/*c*) ratio was determined as 20 : 80, and subsequent irradiation of the same sample with 630 nm resulted in virtually quantitative recovery of (*o*)-**1** (>99%).

The molecular structures of the ring-open and -closed isomers were confirmed by single crystal X-ray diffraction analysis. Two batches of suitable crystals of (*o*)-1 were grown by slow evaporation of solutions in CH_2_Cl_2_/MeOH and CH_2_Cl_2_/EtOH. In the crystals obtained from the former solvent mixture, the calix [4]pyrrole unit is present in a 1,3-alternate conformation and two co-crystallized molecules of MeOH are hydrogen bonded with a pair of pyrrole NH atoms [N(H)⋯O distances between 3.040–3.234 Å, Fig. 2A].^16^ Interestingly, when EtOH instead of MeOH was used, the calix[4]pyrrole displayed a partial cone conformation (Fig. 2B). Also here hydrogen bonding with two solvent molecules was seen, however, now one interacted with three NH atoms [N(H)⋯O distance between 3.136–3.152 Å], while the other had only a single hydrogen bonding interaction [N(H)⋯O distance: 2.961 Å]. Calix[4]pyrrole is conformationally flexible and known to interconvert between 1,2-alternate, 1,3-alternate, and (partial) cone conformations in solution.^8,17,18^ Nevertheless, to the best of our knowledge, the partial cone has been rarely observed in solid state structures.^19^

**Fig. 2:**
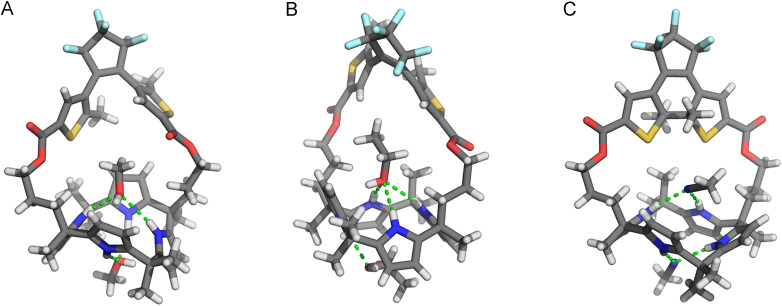
Stick representations of (A) (*o*)-1·(MeOH)_2_, (B) (*o*)-1·(EtOH)_2_, and (C) (*S*,*S*)-(*c*)-1·(MeCN)_2_ as found in the crystal structures; Only one of the enantiomers that is present in the unit cell is depicted.

The DTE photoswitch adopts a parallel conformation in both single crystal X-ray structures of (*o*)-1. It should be noted, however, that since the photoinduced ring-closing of DTE (*vide supra*) is allowed only in the anti-parallel conformation according to the Woodward–Hoffman rules,^12^ both conformers must be in equilibrium in solution.^18^

Single crystals of (*c*)-1 were obtained by 300 nm irradiation of a solution of (*o*)-1 in MeCN, which was first heated and then allowed to cool down (to −7 °C). In this case, the calix[4]pyrrole unit adopted a 1,2-alternate conformation (Fig. 2C), whereas the ring-closed isomer of DTE was present as (*S*,*S*)- and (*R*,*R*)-enantiomer. Again, two co-crystallized solvent molecules (*i.e.*, MeCN) were hydrogen bonded by a pair of pyrrole NH atoms [N(H)⋯N distance: 3.100, 3.104 Å and 2.266, 2.361 Å, respectively]. Clearly different from the open-ring structures is that there is less space available for the solvent molecule in the binding site provided by the strap. Hence, it was envisioned that photo-isomerization would affect the binding affinity for halide ions.

Titrations were performed by ^1^H NMR spectroscopy using the tetrabutylammonium salts in MeCN-*d*_3_. Addition of Cl^−^, Br^−^, and I^−^ to (*o*)-1 led to substantial downfield displacement of the pyrrole-NH (H_a_) signals (Δ*δ* = 3.56, 3.08, and 1.71 ppm, respectively), whereas the β-pyrrolic CH signals (H_b,c_) shifted upfield (Fig. S15, S16 and S18 in the ESI†). Broadening of these signals was observed at the outset of the titrations up to the addition of 1 equivalent, which may be related to interchange between an unbound alternate and a bound cone conformation.^17^ In the case of Cl^−^, the spectrum did not change further beyond the addition of 1 equivalent and the bound and unbound species showed distinct sets of signals, illustrative of strong binding and slow exchange kinetics on the NMR timescale. This exchange was fast for Br^−^ and I^−^, as could be deduced from the gradual chemical shift change, where saturation was nearly reached in the presence of 1 equivalent of the former anion. To quantify the binding affinity for Cl^−^ and Br^−^, isothermal titration calorimetry (ITC) was used (Fig. S24, S25 in the ESI†). The binding of these anions with (*o*)-1 appeared to be driven by enthalpy (Δ*H* = −7.3 kcal mol^−1^ for Cl^−^ and −4.6 kcal for Br^−^) with a small entropic contribution (Δ*S* = 0.32 cal mol^−1^ for Cl^−^ and 2.7 cal for Br^−^). The association constants are given in Table 1 and reveal the strongest binding for Cl^−^, in line with what is reported for other strapped calix[4]pyrrole receptors.^9,10^ To determine the binding constant for I^−^, the ^1^H NMR titration data was fitted to a 1 : 1 binding model using HypNMR software,^20^ affording a much lower constant than determined for Cl^−^ and Br^−^ (see Table 1 and Fig. S19 in the ESI†).

**Table 1:** Association constants (*K*_a_) of halide anions in MeCN

Anion	*K*_a_(*o*)^a^ (M^−1^)	*K*_a_(*c*)^b^ (M^−1^)	*K*_a_(*o*)/*K*_a_(*c*)
Cl^−^	2.6 × 10^5^	1.2 × 10^5^	2.2
Br^−^	8.8 × 10^3^	54	163
I^−^	23	<1	—

a As determined by ITC titrations for Cl^−^ and Br^−^ and by ^1^H NMR titrations for I^−^; Fitting of the ^1^H NMR titration data for Br^−^ by HypNMR^20^ afforded *K*_a_ = 1.0 × 10^4^ M^−1^ (Fig. S17 in the ESI).

b As determined by ^1^H NMR competitive titrations to PSS_300_ (*c*)-1/(*o*)-1 mixtures.

Binding to the ring-closed isomer was also studied by ^1^H NMR spectroscopic titrations, now using the 80 : 20 (*c*)-1/(*o*)-1 PSS_300_ mixture. Upon addition of Cl^−^, similar chemical shift changes as for the ring-open isomer were observed and association/dissociation kinetics were again slow on the NMR timescale (Fig. S20 in the ESI†). In the presence of less than 1 equivalent, the chloride-bound (*c*/*o*) ratio was smaller than the unbound one [for example for 0.16 equiv. (*c*)-1⊂Cl^−^/(*o*)-1⊂Cl^−^ 60 : 40], which indicates weaker binding to the ring-closed than to the ring-open isomer. Determination of the (*c*/*o*) ratio at different equivalents by ^1^H NMR signal integration allowed calculation of a relative binding constant for (*c*)-1, and revealed it to be roughly half of the value determined for (*o*)-1 (see Table 1 and the ESI†).

Stepwise addition of Br^−^ to the PSS_300_ mixture showed fast exchange on the NMR timescale between bound and unbound species. In addition, the ^1^H NMR signals of the minor fraction of (*o*)-1 shifted first, after which the signals of (*c*)-1 followed (Fig. S21 in the ESI†). Fitting of the data (Fig. S22 in the ESI† for details) strikingly revealed that binding to the photogenerated ring-closed isomer is about 160-fold weaker than to the ring-open isomer, which represents the largest swing in affinity for Br^−^ reported so far.^4,5^ Interestingly, alternative addition of I^−^ (up to 45 equivalents) did not cause noteworthy chemical shift changes of the signals belonging to (*c*)-1, revealing that binding was negligible (Fig. S23 in the ESI†).

Overall, the change in binding affinity upon photoisomerization of DTE-strapped calix[4]pyrrole 1 is thus highly dependent on the size of halide ion used, which is a unique observation. While the substrates are geometrically similar (they differ only slightly in their radii), the binding affinity for Cl^−^ is only mildly affected by photoinduced ring-closing, whereas that for Br^−^ is drastically reduced. This results in an almost 75-fold enhancement of the anion selectivity coefficient *k*_Cl/Br_ from 30 to 2.2 × 10^3^.

In conclusion, we have synthesized a DTE-strapped calix[4]pyrrole receptor that can be switched effectively between ring-open and -closed isomers using 300/630 nm light. This process brings about a subtle change in the size of the binding pocket, which has minor influence on the affinity for Cl^−^, but majorly affects the binding of the larger Br^−^ ion, such that Cl^−^/Br^−^ selectivity is largely changed. Such switching of selectivity, in addition to affinity, will provide new opportunities for precise ion separations, as well as the emulation of neuronal networks.

Financial support from the European Research Council (Starting Grant no. 802830 to S. J. W.) and the Dutch Research Council (NWO-ENW, Vidi Grant no. VI.Vidi.192.049 and M Grant no. OCENW.M20.306 to S. J. W.) is gratefully acknowledged. We thank Dr Karthick B. Sai Sankar Gupta and Alfons Lefeber for help with NMR experiments.

## Conflicts of interest

There are no conflicts to declare.

## Supplementary Material

CC-059-D3CC02264A-s001

CC-059-D3CC02264A-s002
