# Supplementary material for: Photoswitching of halide-binding affinity and selectivity in dithienylethene-strapped calix[4]pyrrole
Source: Chem Commun (Camb). 2023 Jun 22;59(56):8688–91. doi: 10.1039/d3cc02264a (PMC10334363; doi:10.1039/d3cc02264a)
Supplement: CC-059-D3CC02264A-s001 [file CC-059-D3CC02264A-s001.pdf]

Electronic Supplementary Material (ESI) for ChemComm.
This journal is © The Royal Society of Chemistry 2023

ELECTRONIC SUPPLEMENTARY INFORMATION (ESI)

Photoswitching of halide-binding affinity and selectivity in dithienylethene-strapped calix[4]pyrrole

David Villarón,[a] Guido E. A. Brugman,[a] Maxime A. Siegler,[b] and Sander J. Wezenberg*,[a]

[a] *Leiden Institute of Chemistry, Leiden University,*
Einsteinweg 55, 2333 CC Leiden, The Netherlands

[b] *Department of Chemistry, Johns Hopkins University,*
3400 N. Charles St., Baltimore, MD, 21218, United States

Email: s.j.wezenberg@lic.leidenuniv.nl

Table of contents

Experimental section S2

^{1}H, ^{13}C and ^{19}F NMR spectra of new and title compounds S5

UV-Vis photoisomerization experiment S14

^{1}H NMR photoisomerization experiment S15

Single crystal X-Ray crystallography S16

^{1}H NMR titration experiments S22

ITC titration experiments S31

References S33

Experimental section

General methods and materials:

THF, MeCN, and Et_2O were dried using a Pure Solve 400 solvent purification system from Innovative Technology. Dry DMF was acquired from Acros Organics and DMSO-d_6, MeCN-d_3, acetone-d_6 and methanol-d_4 were purchased from Eurisotop. DMSO-d_6 was stored under N_2 over molecular sieves (4Å). Degassing of solvents was carried out by purging with N_2 for 30 min, unless noted otherwise. Compounds (*o*)-**2**[1] and **5**[2] were prepared using procedures reported in the literature. All other chemicals were commercial products and were used without further purification. Column chromatography was performed using silica gel (SiO_2) purchased from Screening Devices BV (Pore diameter 55-70 Å, surface area 500 m^2g^{-1}). Thin-layer chromatography (TLC) was carried out on aluminum sheets coated with silica 60 F254 and neutral aluminum oxide obtained from Merck. Compounds were visualized with UV light (254 nm). Melting points were determined using a Büchi M560 apparatus. ^{1}H, ^{13}C and ^{19}F NMR spectra were recorded on Bruker AV 400 and Bruker 500 Ultra Shield instruments at 298 K unless indicated otherwise. Chemical shifts (δ) are denoted in parts per million (ppm) relative to residual protiated solvent (DMSO-d_6: for ^{1}H detection, δ = 2.50 ppm; for ^{13}C detection, δ = 39.52 ppm; MeCN-d_3: for ^{1}H detection, δ = 1.94 ppm; for ^{13}C detection, δ = 1.32 and 118.26 ppm; acetone-d_6 for ^{1}H detection, δ = 2.05 ppm; for ^{13}C detection, δ = 29.84 and 206.26 ppm; methanol-d_3 for ^{1}H detection, δ = 3.31 ppm; for ^{13}C detection, δ = 49.0 ppm. The splitting pattern of peaks is designated as follows: s (singlet), d (doublet), dd (double doublet), t (triplet), m (multiplet), and br (broad). IR spectra were recorded on a Perkin Elmer Spectrum Two FT-IR spectrometer. The wavenumber (ν) is in units of reciprocal centimeters (cm^{-1}) and the intensity is designated as follows: s (strong), m (medium), w (weak), very w (very weak), br (broad), and sh (shoulder). High-resolution mass spectrometry (ESI-MS) was performed on a Thermo Scientific Q Exactive HF spectrometer with ESI ionization. UV-Vis spectra were recorded on an Agilent Cary 8454 spectrometer using 1 cm or 1 mm quartz cuvettes. ITC measurements were performed using a MicroCal VP-ITC MicroCalorimeter. Irradiation of UV-Vis and NMR samples was carried out using Thorlab model M300F2 (4.8 mW) and 630E (7.2 mW) LEDs positioned at a distance of 1 cm.

4,4'-(Perfluorocyclopent-1-ene-1,2-diyl)bis(5-methylthiophene-2-carboxylic acid) [(*o*)-3]: *n*-Butyllithium (3.6 mL, 1.6 M solution in hexanes) was added dropwise over 20 min to a stirred solution of compound (*o*)-**2** (1.0 g, 2.3 mmol) in dry Et_2O (110 mL) at rt under N_2 atmosphere. The resulting mixture was cooled to –78 °C and purged with CO_2 (g) for 10 min, after which it was allowed to warm to rt and stirred for a further 3 h. The mixture was treated with 0.5 M aqueous HCl solution (200 mL) and the layers were separated. The aqueous layer was extracted with Et_2O (3 × 100 mL) and the combined organic layers were dried over Na_2SO_4 and concentrated. Purification by column chromatography (SiO_2; CH_2Cl_2/AcOH 95:5) afforded (*o*)-**3** as white solid (0.81 g, 78%); R_f = 0.52 (SiO_2; CH_2Cl_2/AcOH 95:5); m.p. >260 °C (decomp); ^{1}H NMR (400 MHz, methanol-d_4): δ = 7.72 (s, 2H), 1.99 (s, 6H) ppm; ^{13}C NMR (100 MHz, methanol-d_4): δ = 164.1, 150.4, 134.6, 133.9, 126.5, 117.3, 14.9 ppm; ^{19}F NMR (470 MHz, methanol-d_4): δ = –111.9, –133.5 ppm; IR (ATR): ν = 2820 (br. m), 1679 (s), 1549 (m), 1472 (m), 1416 (w), 1335 (w), 1265 (s), 1190 (s), 1127 (s), 1038 (s), 983 (m), 898 (m), 774 (m, sh), 743 (m), 666 (m), 567 (m), 540 (m), 512 (m), 477 (w, cm^{-1}); HRMS (ESI) *m*/*z*: 456.9995 ([M+H]$^+$, calcd for $C_{17}H_{11}F_6O_4S_2^+$: 456.9998.

Dithienylethene-linked bis(dipyrromethane) [(*o*)-4]:
A mixture of compound (*o*)-**3** (0.50 g, 1.1 mmol), TBTU (0.70 g, 2.2 mmol), and DIPEA (0.42 mL, 2.4 mmol) in dry DMF (2.5 mL) was stirred for 20 min at rt. Compound **5** (0.60 g, 2.7 mmol) in dry DMF (2.5 mL) was added and the mixture was stirred for a further 17 h at rt. It was then diluted with CH_2Cl_2 (25 mL) and washed with 0.5 M aqueous HCl solution (2 × 30 mL), saturated aqueous $NaHCO_3$ solution (2 × 30 mL), and Brine (30 mL). The organic phase was dried over Na_2SO_4 and concentrated. Purification by column chromatography (SiO_2; heptane/*i*PrOH 95:5) afforded (*o*)-**4** as brown solid (0.67 g, 71%); R_f = 0.86 (SiO_2; pentane/*i*PrOH 90:10); m.p. 154.0 – 156.7 °C; ^{1}H NMR (400 MHz, DMSO-d_6): δ = 10.30 (s, 4H), 7.76 (s, 2H), 6.59 – 6.53 (m, 4H), 5.87 – 5.82 (m, 4H), 5.74 – 5.68 (m, 4H), 4.15 (t, *J* = 6.6 Hz, 4H), 2.05 – 1.98 (m, 4H), 1.97 (s, 6H), 1.53 (s, 6H), 1.51 – 1.43 (m, 4H) ppm; ^{13}C NMR (100 MHz, DMSO-d_6): δ = 160.5, 149.3, 138.0, 132.7, 131.5, 124.5, 116.6, 106.3, 103.8, 65.7, 38.3, 36.7, 25.1, 24.0, 14.0 ppm; ^{19}F NMR (470 MHz, DMSO-d_6): δ= –109.4, –130.7 ppm; IR (ATR): ν = 3374 (w), 2926 (w), 1702 (m), 1552 (w) 1467 (m), 1338 (w), 1271 (s), 1191 (s), 1117 (s), 1068 (s), 1032 (m, sh), 984 (m), 883 (m), 793 (m), 718 (s), 567 (m), 540 (m, cm^{-1}); HRMS (ESI) *m*/*z*: 857.2621 ([M+H]$^+$, calcd for $C_{43}H_{43}N_4F_6O_4S_2^+$: 857.2624.

Dithienylethene-strapped calix[4]pyrrole [(*o*)-1]:

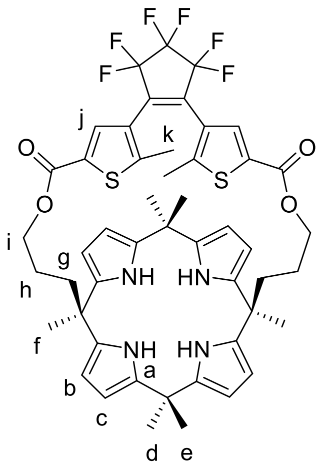

$BF_3.OEt_2$ (80 μL, 0.64 mmol) was added dropwise to a stirred solution of compound (*o*)-**4** (1.1 g, 1.3 mmol) in acetone (630 mL) at rt and after 1 h the mixture was treated with Et_3N (18 mL) and concentrated. Purification by column chromatography (SiO_2; pentane/EtOAc 9:1) afforded (*o*)-**1** (0.26 g, 21 %) as white solid; R_f = 0.53 (SiO_2; pentane/EtOAc 9:1); m.p. >250 °C (decomp); ^{1}H NMR (500 MHz, acetone-d_6, assignment is based on COSY and NOESY spectra): δ = 8.41 (br. s, 4H; H_a), 7.79 (s, 2H; H_j), 5.72 (dd, J = 3.4, 2.7 Hz, 4H; H_c), 5.69 (dd, J = 3.4, 2.7 Hz, 4H; H_b), 4.22 (t, J = 5.6 Hz, 4H; H_i), 2.36 (s, 6H; H_k), 1.96 – 1.90 (m, 4H; H_g), 1.59 – 1.52 (m, 4H; H_h), 1.54 (s, 6H; H_d), 1.50 (s, 6H; H_f), 1.45 (s, 6H; H_e) ppm; ^{13}C NMR (125 MHz, acetone-d_6): δ = 161.2, 149.7, 139.5, 137.9, 134.3, 132.6, 125.9, 104.1, 103.0, 65.6, 39.0, 38.8, 35.8, 31.5, 27.2, 25.4, 24.6, 15.1 ppm; ^{19}F NMR (470 MHz, DMSO-d_6): δ = –110.9, –132.1 ppm; IR (ATR): ν = 3441 (w), 2924 (m), 2854 (m, sh), 1716 (s), 1551 (very w), 1457 (w), 1363 (w), 1334 (w), 1273 (m, sh), 1248 (s), 1187 (s, sh), 1120 (s), 1083 (s), 1062 (sh, m), 981 (m), 891 (w), 871 (w, sh),771 (s), 743 (s), 707 (m, sh), 663 (w), 567 (w), 538 (m), 511 (m), 473 (w, cm^{-1}); HRMS (ESI) *m/z*: 937.3245 ($[M+H]^+$, calcd for $C_{49}H_{51}F_6N_4O_4S_2^+$: 937.3250.

^{1}H, ^{13}C and ^{19}F NMR spectra of new and title compounds

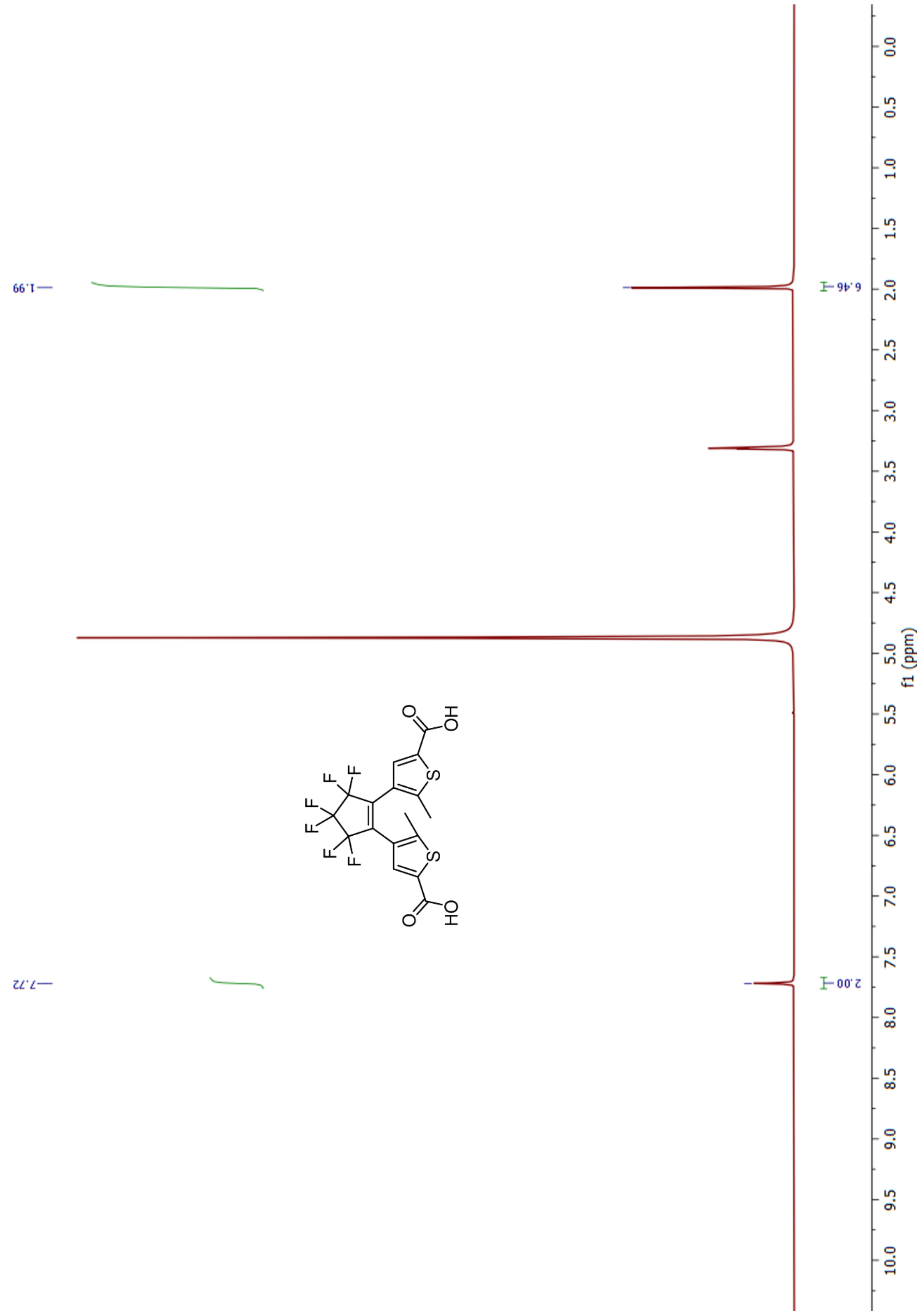

Figure S1. 400 MHz ^{1}H NMR spectrum of (*o*)-**3** measured at 298 K in methanol-d_4.

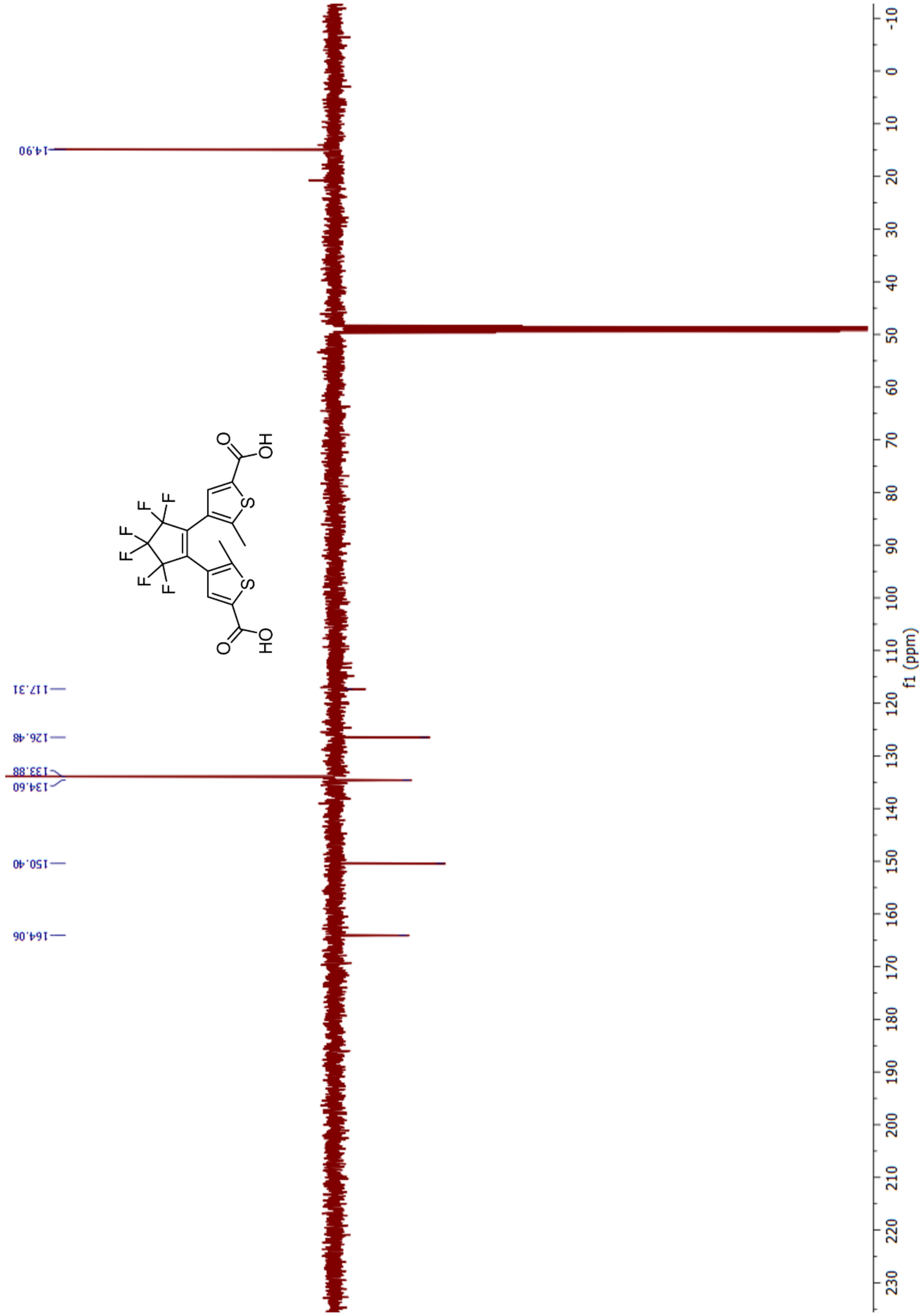

Figure S2. 100 MHz ^{13}C NMR APT spectrum of (*o*)-**3** measured at 298 K in methanol-d_4: CH and CH_3 signals positive and quaternary carbon and CH_2 signals negative.

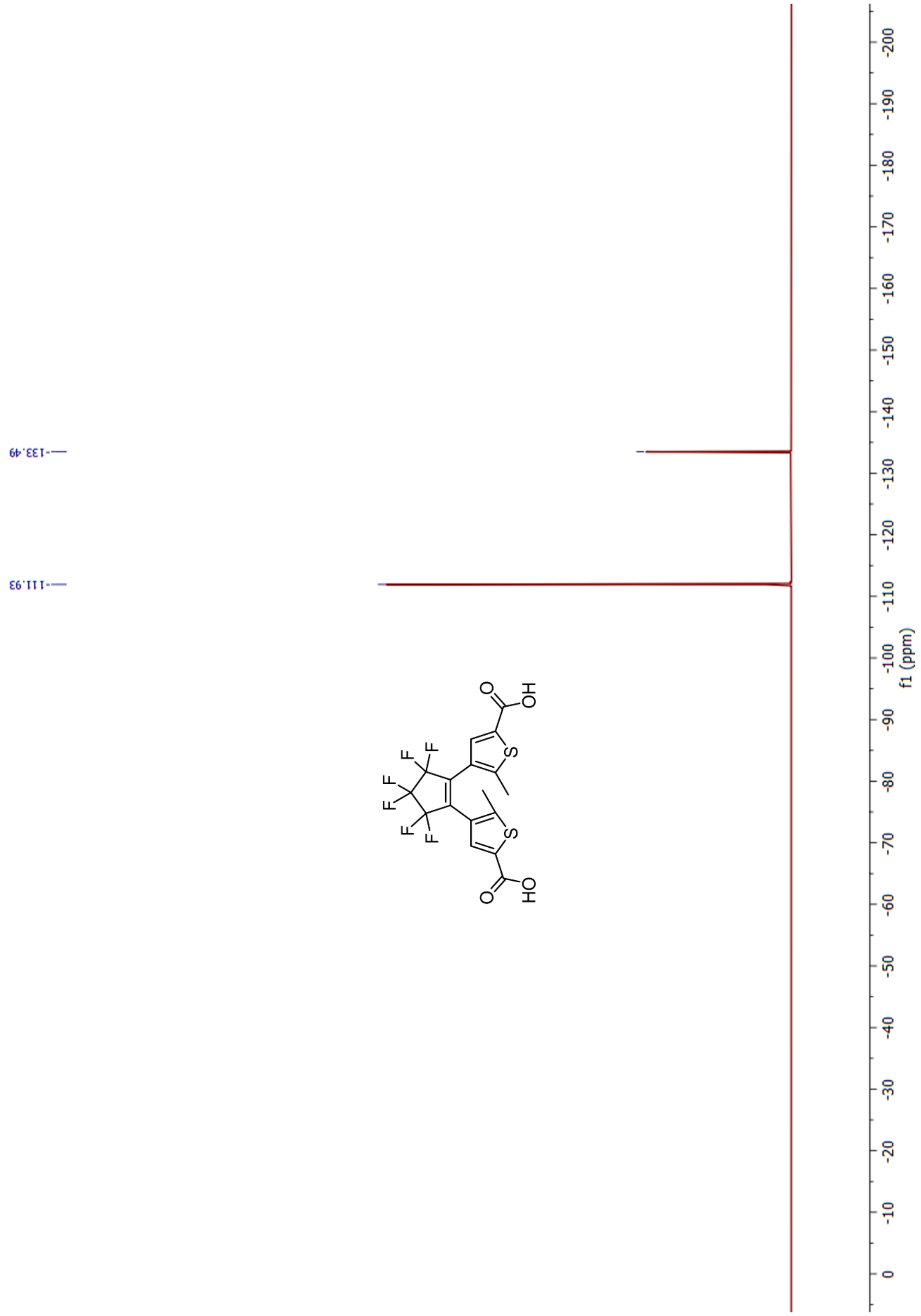

Figure S3. 470 MHz ^{19}F NMR spectrum of (*o*)-**3** measured at 298 K in methanol-d_4.

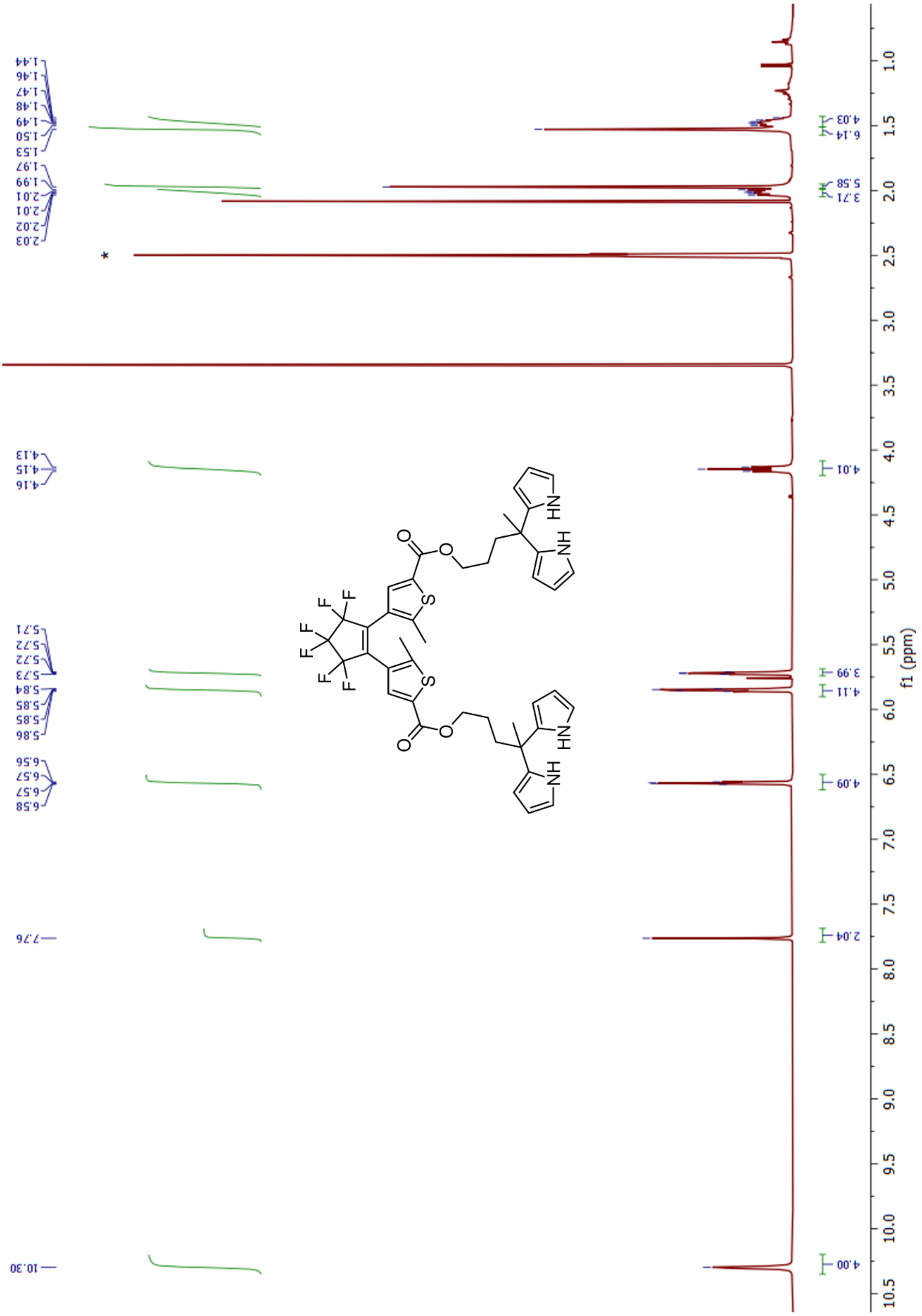

Figure S4. 400 MHz ^{1}H NMR spectrum of (*o*)-**4** measured at 298 K in DMSO-d_6.

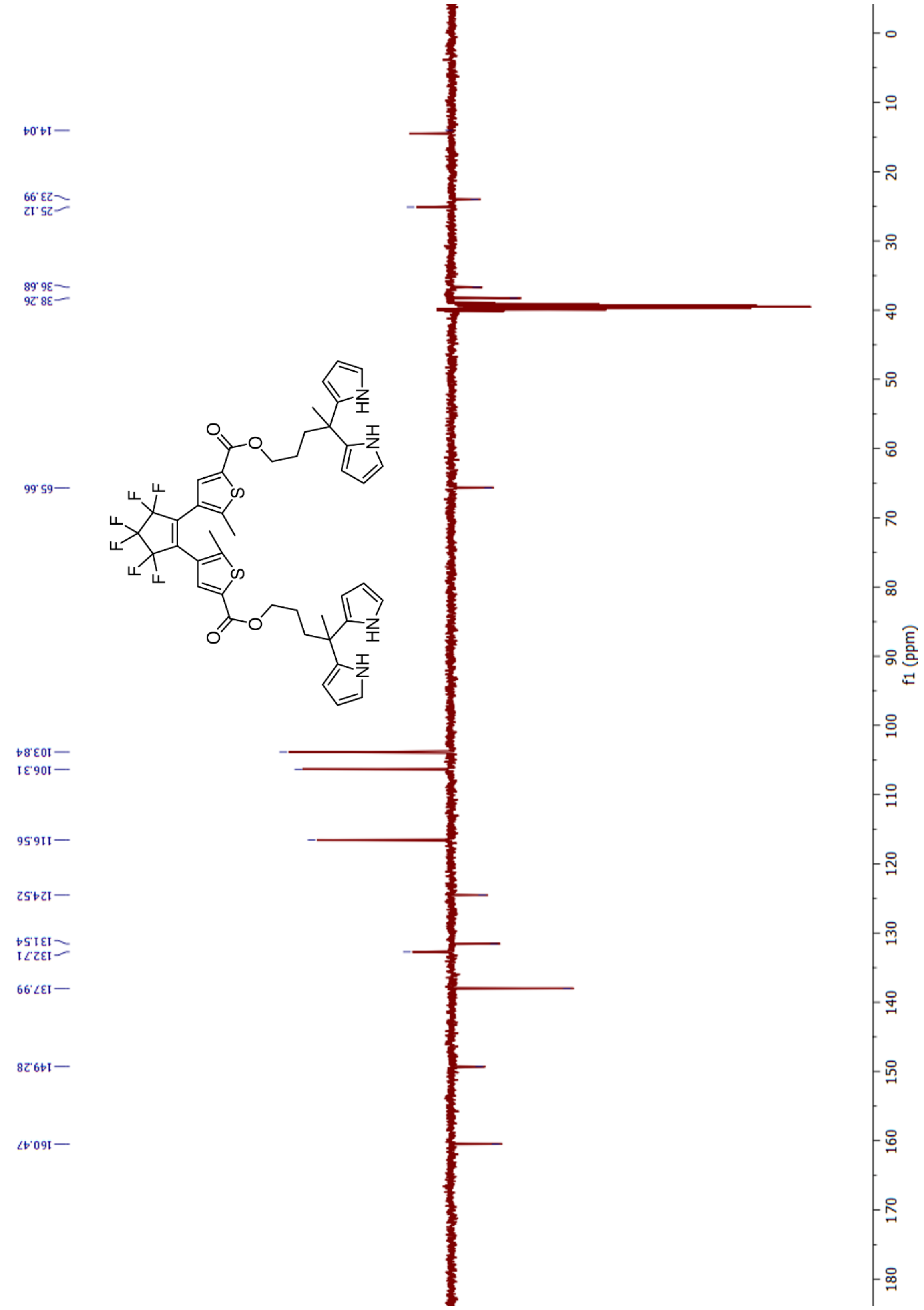

Figure S5. 100 MHz ^{13}C NMR APT spectrum of (*o*)-**4** measured at 298 K in DMSO-d_6 :CH and CH_3 signals positive and quaternary carbon and CH_2 signals negative.

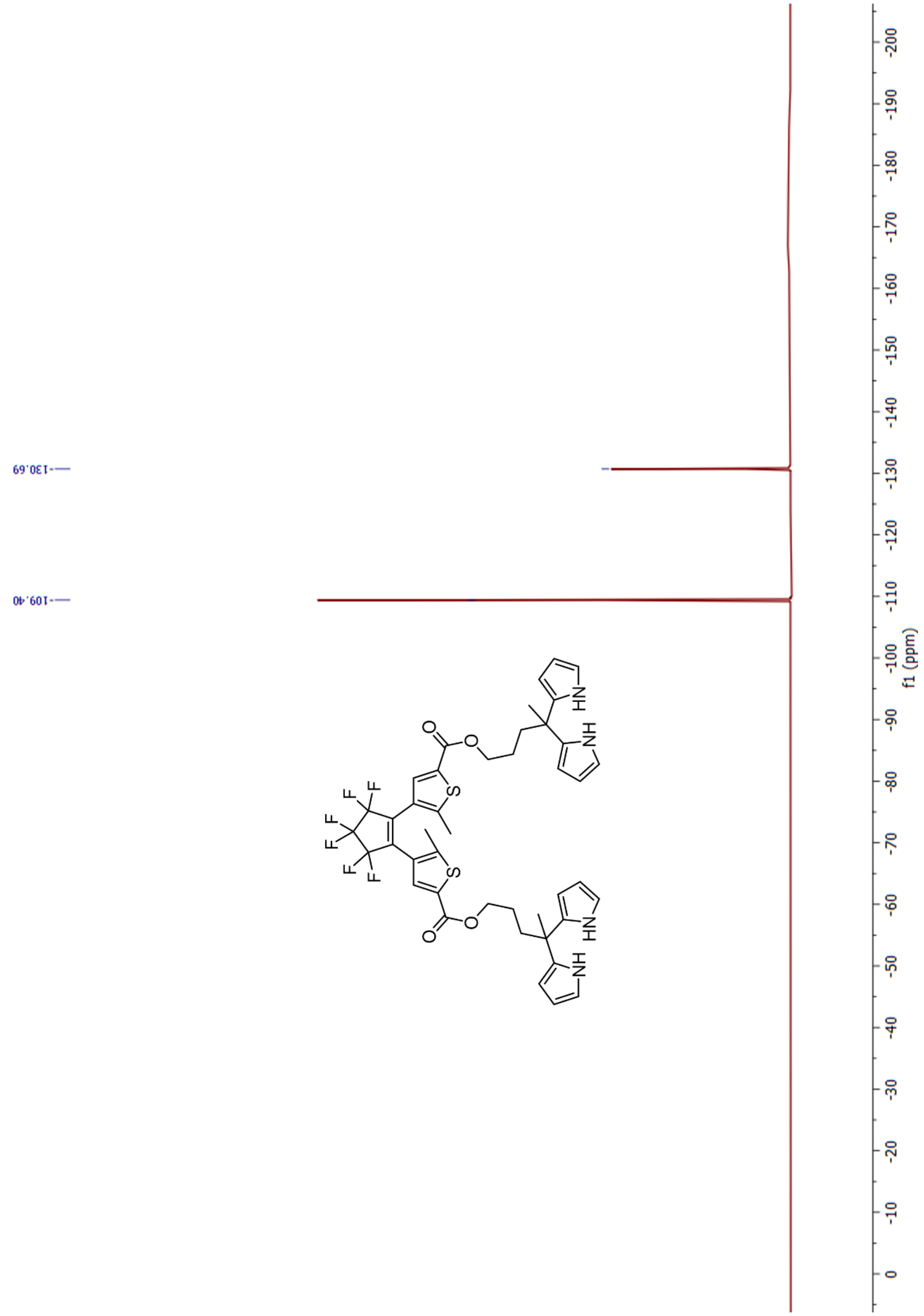

Figure S6. 470 MHz ^{19}F NMR spectrum of (*o*)**-4** measured at 298 K in DMSO-d_6.

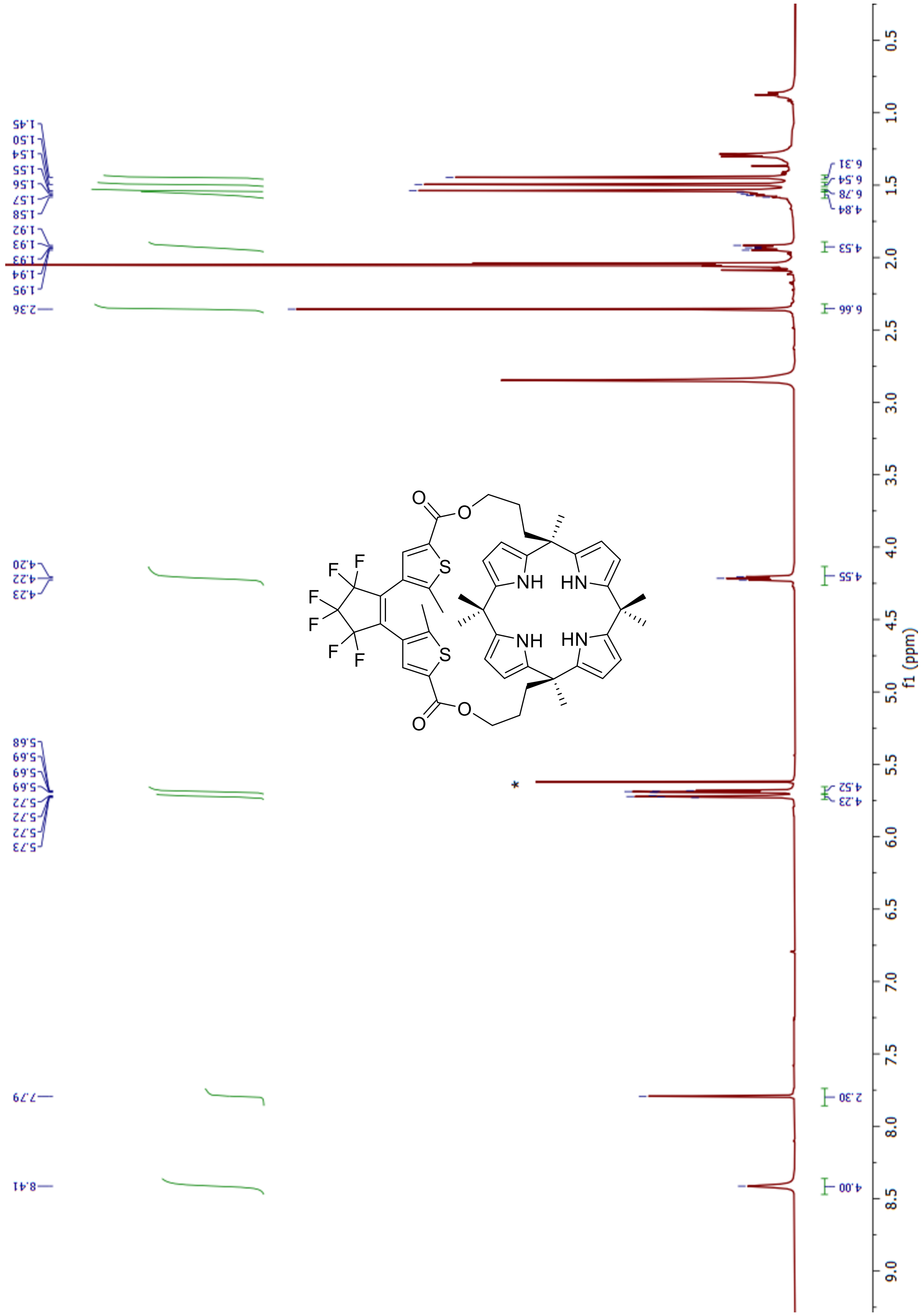

Figure S7. 500 MHz ^{1}H NMR spectrum of (*o*)-**1** measured at 298 K in acetone-d_6; *CH_2Cl_2.

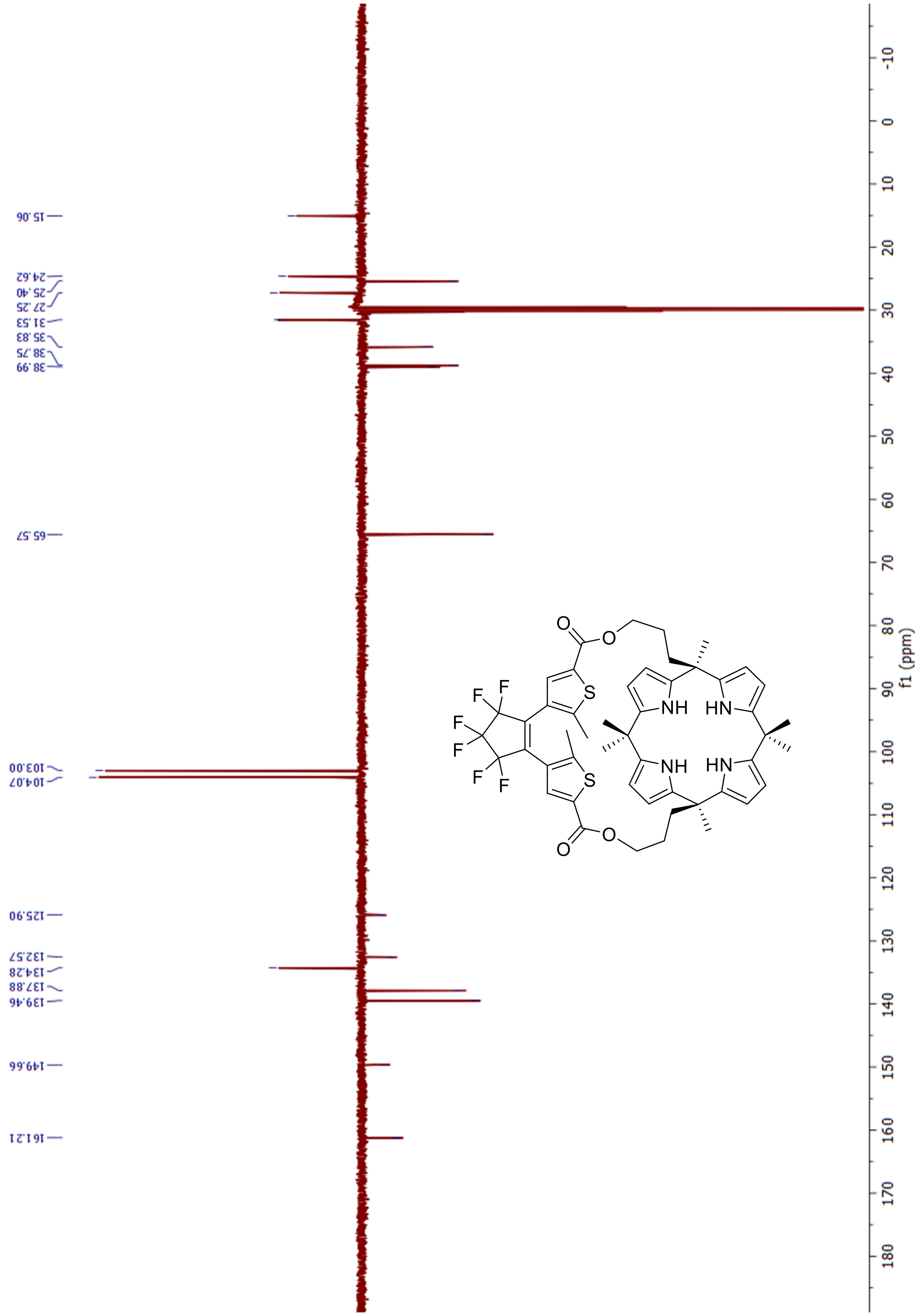

Figure S8. 125 MHz ^{13}C NMR APT spectrum of (*o*)-**1** measured at 298 K in acetone-d_6 :CH and CH_3 signals positive and quaternary carbon and CH_2 signals negative.

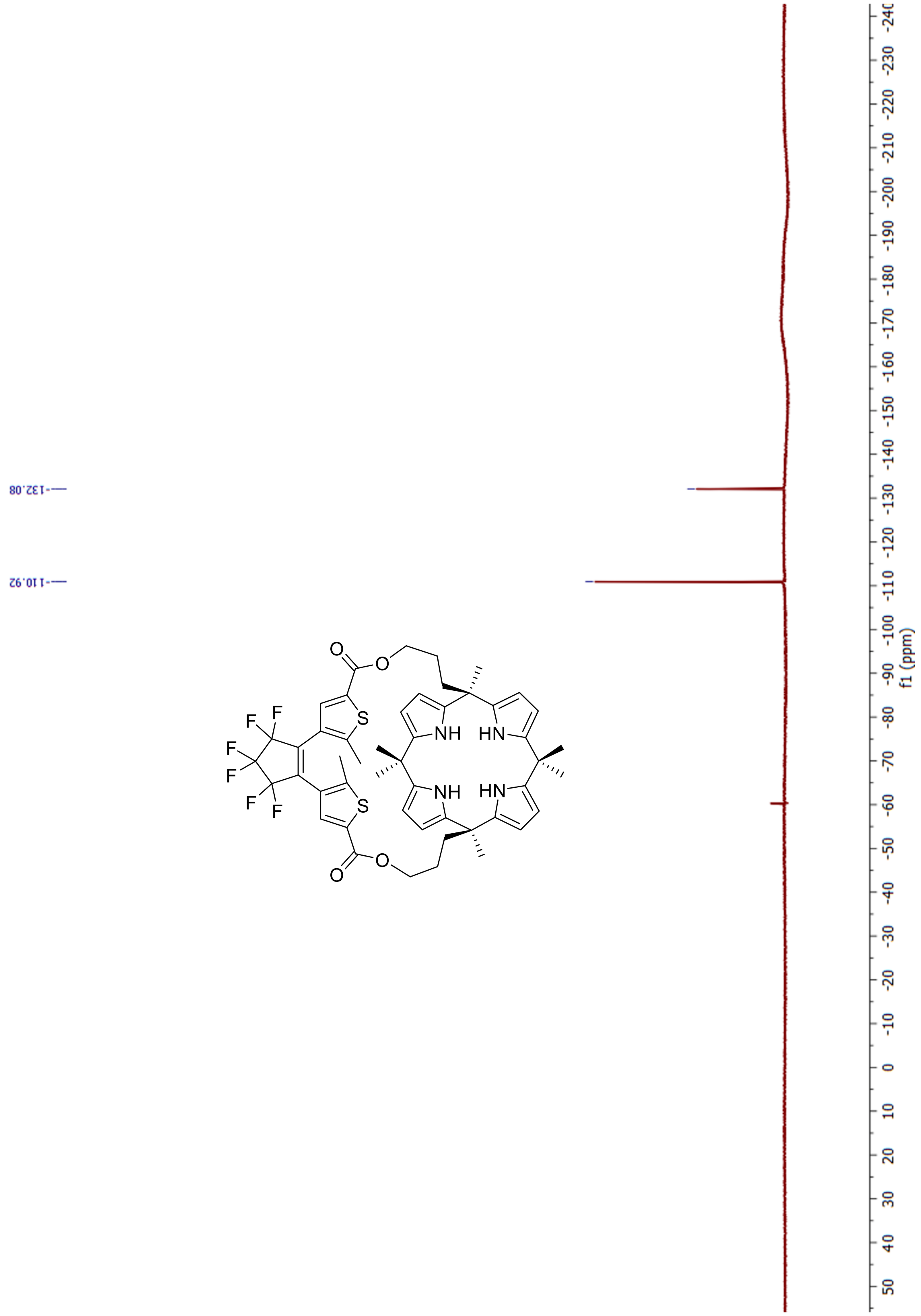

Figure S9. 470 MHz ^{19}F NMR spectrum of (*o*)-**1** measured at 298 K in DMSO-d_6.

UV-Vis photoisomerization experiment

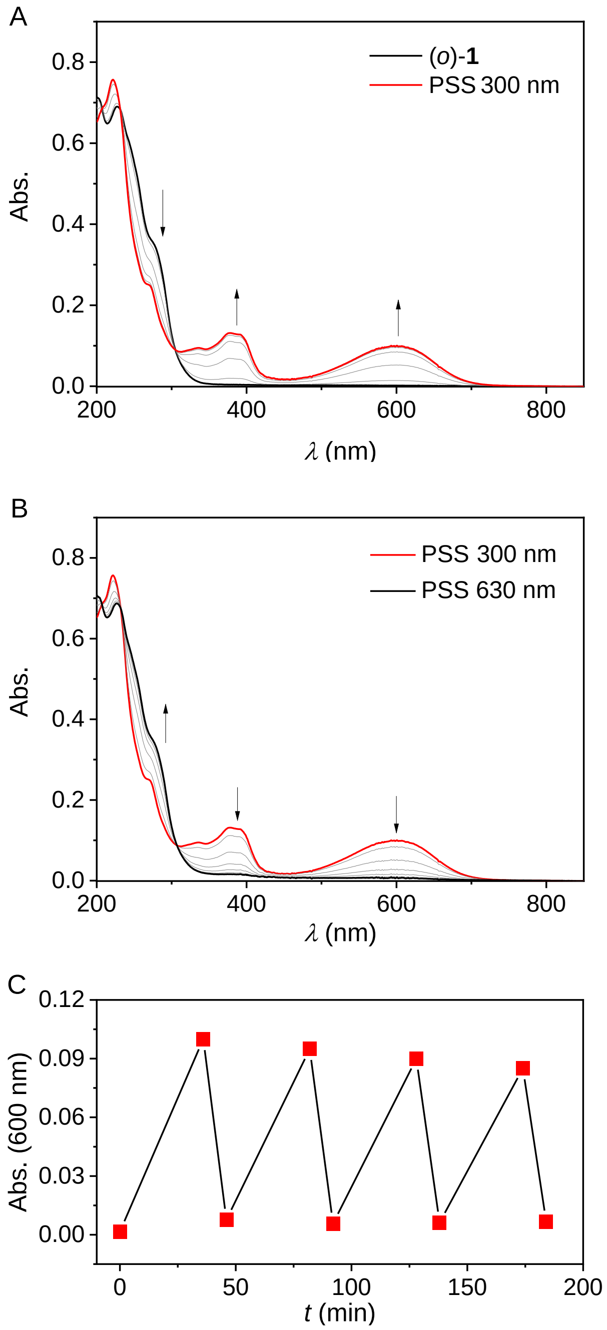

Figure S10. (A) UV-Vis spectral changes of (*o*)-**1** (2.1×10^{-5} M in MeCN) upon 300 nm irradiation for 36 min and (B) consecutive 630 nm irradiation for 10 min showing an isosbestic point at $\lambda = 305$ nm. (C) Plot of the change in absorption at 600 nm upon multiple 300/630 switching cycles. The slight decrease in absorbance noted upon multiple 300/630 nm irradiation cycles (~5% after each cycle) could be most likely ascribed to an unidentified photoinduced side reaction of calix[4]pyrrole upon prolonged irradiation at 300 nm.

^{1}H NMR photoisomerization experiment

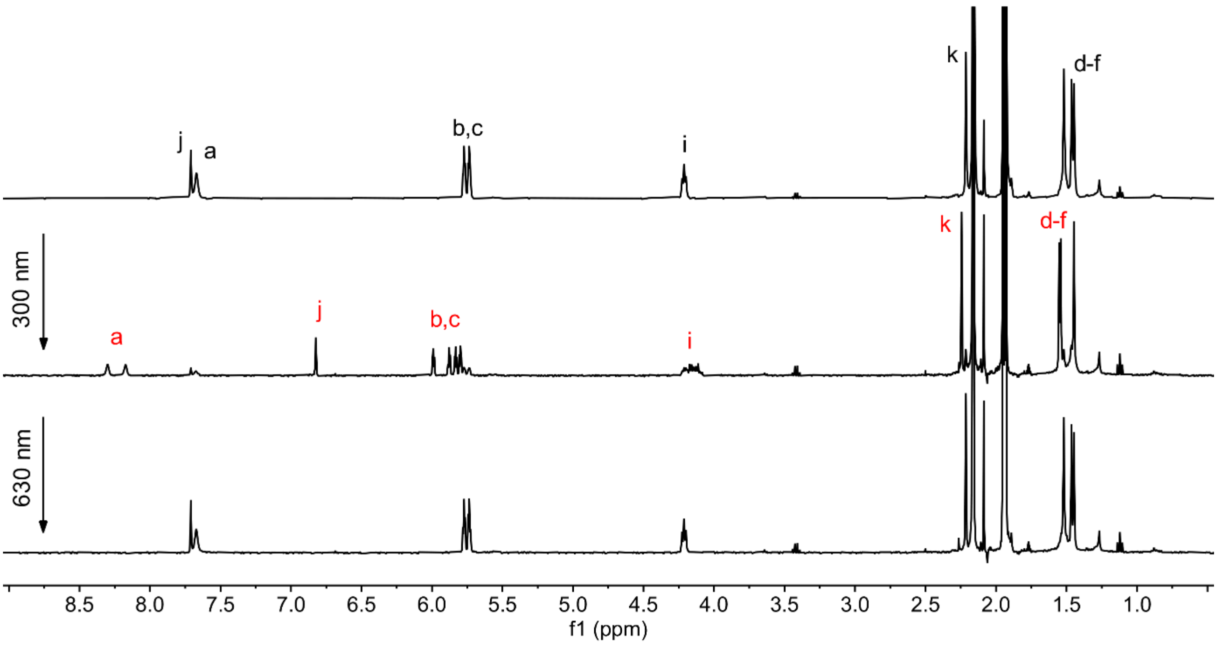

Figure S11. ^{1}H NMR Spectrum (400 MHz, 293 K) of (*o*)-**1** in degassed MeCN-d_3 (2.0 mM) (top) and spectral changes after irradiation with 300 nm light for 5 h (middle), followed by irradiation with 630 nm light for 30 min (bottom) at 20 °C. For determination of the PSS ratios, the integrals of the pyrrole NH (H_a) and aromatic thiophene (H_j) signals were averaged giving a 20:80 (*o*/*c*) ratio at 300 nm and 99:1 (*o*/*c*) at 630 nm.

Single crystal X-Ray crystallography

Single crystals of (*o*)-1 obtained by slow evaporation of a solution in CH_2Cl_2/MeOH:

All reflection intensities were measured at 110(2) K using a SuperNova diffractometer (equipped with Atlas detector) with Mo $K\alpha$ radiation (λ = 0.71073 Å) under the program CrysAlisPro (Version CrysAlisPro 1.171.39.29c, Rigaku OD, 2017). The same program was used to refine the cell dimensions and for data reduction. The structure was solved with the program SHELXS-2018/3 (Sheldrick, 2018) and was refined on F^2 with SHELXL-2018/3 (Sheldrick, 2018). Numerical absorption correction based on gaussian integration over a multifaceted crystal model was applied using CrysAlisPro. The temperature of the data collection was controlled using the system Cryojet (manufactured by Oxford Instruments). The H atoms were placed at calculated positions (unless otherwise specified) using the instructions AFIX 23, AFIX 43, AFIX 137 or AFIX 147 with isotropic displacement parameters having values 1.2 or 1.5 U_{eq} of the attached C or O atoms. The H atoms attached to N30, N38, N45 and N53 were found from difference Fourier maps, and their coordinates were refined pseudofreely using the DFIX instruction in order to keep the N–H distances within an acceptable range. The H atom attached to O1S' (minor component of the disordered lattice methanol molecule) could not be retrieved from difference Fourier map.

The structure is partly disordered. The asymmetric contains two sites occupied by disordered lattice solvent molecules. One site is occupied by one lattice MeOH solvent molecule which is disordered over two orientations (occupancy factor of the major component: 0.641(5)). The second site is occupied by a disordered mixture of lattice MeOH (91.4%) : CH_2Cl_2 (8.6%) solvent molecules.

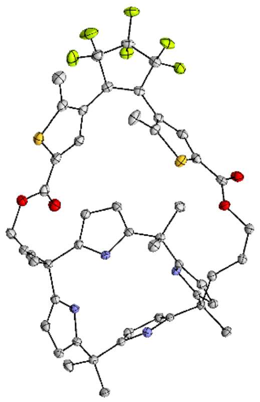

Figure S12. Displacement ellipsoid plot (50% probability level) of (*o*)-**1** (1,3-alternate) at 110(2) K. Lattice solvent molecules are omitted for clarity.

Table S1. Crystallographic data for the structure of (*o*)-**1** (1,3-alternate).

	(*o*)-**1** (1,3-alternate)
Crystal data	
Chemical formula	$C_{51}H_{57.47}Cl_{0.17}F_6N_4O_{5.91}S_2$
M_r	1005.26
Crystal system, space group	Triclinic, *P*-1
Temperature (K)	110
a, *b*, *c* (Å)	10.2802 (4), 14.3512 (7), 17.0649 (7)
α, β, γ (°)	91.974 (4), 100.406 (3), 94.069 (4)
V (Å^3)	2467.12 (19)
Z	2
Radiation type	Mo *K*α
μ (mm^{-1})	0.19
Crystal size (mm)	0.22 × 0.14 × 0.05
Data collection	
Diffractometer	SuperNova, Dual, Cu at zero, Atlas
Absorption correction	Gaussian<br>*CrysAlis PRO* 1.171.41.93a (Rigaku Oxford Diffraction, 2020) Numerical absorption correction based on gaussian integration over a multifaceted crystal model Empirical absorption correction using spherical harmonics, implemented in SCALE3 ABSPACK scaling algorithm.
T_{min}, T_{max}	0.768, 1.000
No. of measured, independent and observed [$I > 2\sigma(I)$] reflections	39820, 11350, 8219
R_{int}	0.057
$(\sin \theta/\lambda)_{max}$ (Å^{-1})	0.650
Refinement	
$R[F^2 > 2\sigma(F^2)]$, $wR(F^2)$, S	0.053, 0.131, 1.04
No. of reflections	11350
No. of parameters	682
No. of restraints	78
H-atom treatment	H atoms treated by a mixture of independent and constrained refinement
$\Delta\rho_{max}$, $\Delta\rho_{min}$ (e Å^{-3})	0.47, -0.47

Computer programs: *CrysAlis PRO* 1.171.39.29c (Rigaku OD, 2017), *SHELXS2018*/3 (Sheldrick, 2018), *SHELXL2018*/3 (Sheldrick, 2018), *SHELXTL* v6.10 (Sheldrick, 2008).[3]

Single crystals of (*o*)-1 obtained by slow evaporation of a solution in CH_2Cl_2/EtOH:

All reflection intensities were measured at 110(2) K using a SuperNova diffractometer (equipped with Atlas detector) with Mo *K*α radiation (λ = 0.71073 Å) under the program CrysAlisPro (Version CrysAlisPro 1.171.39.29c, Rigaku OD, 2017). The same program was used to refine the cell dimensions and for data reduction. The structure was solved with the program SHELXS-2018/3 (Sheldrick, 2018) and was refined on F^2 with SHELXL-2018/3 (Sheldrick, 2018). Numerical absorption correction based on gaussian integration over a multifaceted crystal model was applied using CrysAlisPro. The temperature of the data collection was controlled using the system Cryojet (manufactured by Oxford Instruments). The H atoms were placed at calculated positions (unless otherwise specified) using the instructions AFIX 23, AFIX 43, AFIX 137 or AFIX 147 with isotropic displacement parameters having values 1.2 or 1.5 U_{eq} of the attached C or O atoms. The H atoms attached to N31, N38, N45, and N53 were found from difference Fourier maps, and their coordinates were refined pseudofreely using the DFIX instruction in order to keep the N–H distances within an acceptable range.

The structure is partly disordered. The asymmetric unit contains one disordered mixture of lattice solvent molecule EtOH (87.8%) : CH_2Cl_2 (12.2%).

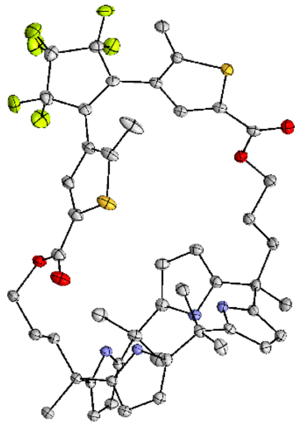

Figure S13. Displacement ellipsoid plot (50% probability level) of (*o*)-**1** (partial cone) at 110(2) K. Lattice solvent molecules are omitted for clarity.

Table S2. Crystallographic data for the structure of (*o*)-**1** (partial cone).

	(*o*)-**1** (partial cone)
Crystal data	
Chemical formula	$C_{49}H_{50}F_6N_4O_4S_2 \cdot 0.122(CH_2Cl_2) \cdot 1.878(C_2H_6O)$
M_r	1033.77
Crystal system, space group	Monoclinic, $P2_1/c$
Temperature (K)	110
a, *b*, *c* (Å)	17.4447 (4), 17.5229 (4), 16.5312 (3)
β (°)	91.766 (2)
V (Å^3)	5050.89 (19)
Z	4
Radiation type	Mo $K\alpha$
μ (mm^{-1})	0.19
Crystal size (mm)	0.69 × 0.29 × 0.23
Data collection	
Diffractometer	SuperNova, Dual, Cu at zero, Atlas
Absorption correction	Gaussian<br>*CrysAlis PRO* 1.171.41.93a (Rigaku Oxford Diffraction, 2020) Numerical absorption correction based on gaussian integration over a multifaceted crystal model Empirical absorption correction using spherical harmonics, implemented in SCALE3 ABSPACK scaling algorithm.
T_{min}, T_{max}	0.513, 1.000
No. of measured, independent and observed [$I > 2\sigma(I)$] reflections	59668, 11600, 9764
R_{int}	0.031
$(\sin \theta/\lambda)_{max}$ (Å^{-1})	0.650
Refinement	
$R[F^2 > 2\sigma(F^2)]$, $wR(F^2)$, S	0.038, 0.097, 1.01
No. of reflections	11600
No. of parameters	692
No. of restraints	64
H-atom treatment	H atoms treated by a mixture of independent and constrained refinement
$\Delta\rho_{max}$, $\Delta\rho_{min}$ (e Å^{-3})	0.41, -0.31

Computer programs: *CrysAlis PRO* 1.171.39.29c (Rigaku OD, 2017), *SHELXS2018*/3 (Sheldrick, 2018), *SHELXL2018*/3 (Sheldrick, 2018), *SHELXTL* v6.10 (Sheldrick, 2008).[3]

Single crystals of (*c*)-1 obtained by cooling of a solution in MeCN (to –7 °C):

All reflection intensities were measured at 110(2) K using a SuperNova diffractometer (equipped with Atlas detector) with Mo *K*α radiation (λ = 0.71073 Å) under the program CrysAlisPro (Version CrysAlisPro 1.171.39.29c, Rigaku OD, 2017). The same program was used to refine the cell dimensions and for data reduction. The structure was solved with the program SHELXS-2018/3 (Sheldrick, 2018) and was refined on F^2 with SHELXL-2018/3 (Sheldrick, 2018). Numerical absorption correction based on gaussian integration over a multifaceted crystal model was applied using CrysAlisPro. The temperature of the data collection was controlled using the system Cryojet (manufactured by Oxford Instruments). The H atoms were placed at calculated positions (unless otherwise specified) using the instructions AFIX 23, AFIX 43 or AFIX 137 with isotropic displacement parameters having values 1.2 U_{eq} of the attached C atoms. The H atoms attached to N30, N38, N45 and N53 were found from difference Fourier maps, and their coordinates were refined pseudofreely using the DFIX instruction in order to keep the N–H distances within an acceptable range.

The structure is partly disordered. The C16-C17-C63-C65 fragment and one of the three crystallographically independent lattice MeCN solvent molecules are disordered over two orientations, and the occupancy factors of the major components of the disorder refine to 0.690(5) and 0.605(6), respectively.

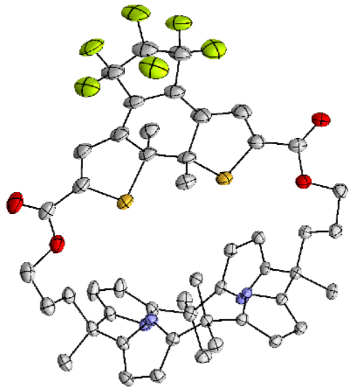

Figure S14. Displacement ellipsoid plot (50% probability level) of (*c*)-**1** at 110(2) K. Disorder and lattice solvent molecules are omitted for clarity.

Table S3. Crystallographic data for the structure of (*c*)-**1**.

	(*c*)-**1**
Crystal data	
Chemical formula	$C_{49}H_{50}F_6N_4O_4S_2{\cdot}3(C_2H_3N)$
M_r	1060.21
Crystal system, space group	Monoclinic, $P2_1/c$
Temperature (K)	110
a, *b*, *c* (Å)	22.7698 (5), 12.6624 (3), 18.6952 (5)
β (°)	91.449 (2)
V (Å^3)	5388.5 (2)
Z	4
Radiation type	Mo $K\alpha$
μ (mm^{-1})	0.17
Crystal size (mm)	0.37 × 0.33 × 0.31
Data collection	
Diffractometer	SuperNova, Dual, Cu at zero, Atlas
Absorption correction	Gaussian<br>*CrysAlis PRO* 1.171.41.93a (Rigaku Oxford Diffraction, 2020) Numerical absorption correction based on gaussian integration over a multifaceted crystal model Empirical absorption correction using spherical harmonics, implemented in SCALE3 ABSPACK scaling algorithm.
T_{min}, T_{max}	0.463, 1.000
No. of measured, independent and observed [$I > 2\sigma(I)$] reflections	80715, 12377, 9930
R_{int}	0.034
$(\sin \theta/\lambda)_{max}$ (Å^{-1})	0.650
Refinement	
$R[F^2 > 2\sigma(F^2)]$, $wR(F^2)$, S	0.049, 0.135, 1.01
No. of reflections	12377
No. of parameters	758
No. of restraints	156
H-atom treatment	H atoms treated by a mixture of independent and constrained refinement
$\Delta\rho_{max}$, $\Delta\rho_{min}$ (e Å^{-3})	0.62, -0.43

Computer programs: *CrysAlis PRO* 1.171.39.29c (Rigaku OD, 2017), *SHELXS2018*/3 (Sheldrick, 2018), *SHELXL2018*/3 (Sheldrick, 2018), *SHELXTL* v6.10 (Sheldrick, 2008).[3]

^{1}H NMR titration experiments

First, a solution of (*o*)-**1** (~2 mM in MeCN-*d*$_3$) was prepared, which was then used to prepare a more concentrated solution of the tetrabutylammonium anion. The solution containing the anion was added stepwise to the solution of (*o*)-**1** and after each addition a ^{1}H NMR spectrum was recorded. For the titrations to (*c*)-**1**, the solution of (*o*)-**1** was irradiated at 300 nm until the PSS ratio was (nearly) reached, before using it to prepare the anion solution. The titration experiment was performed in the dark to avoid back-isomerization from (*c*)-**1** to (*o*)-**1**.

Titration of NBu_4Cl to (*o*)-1:

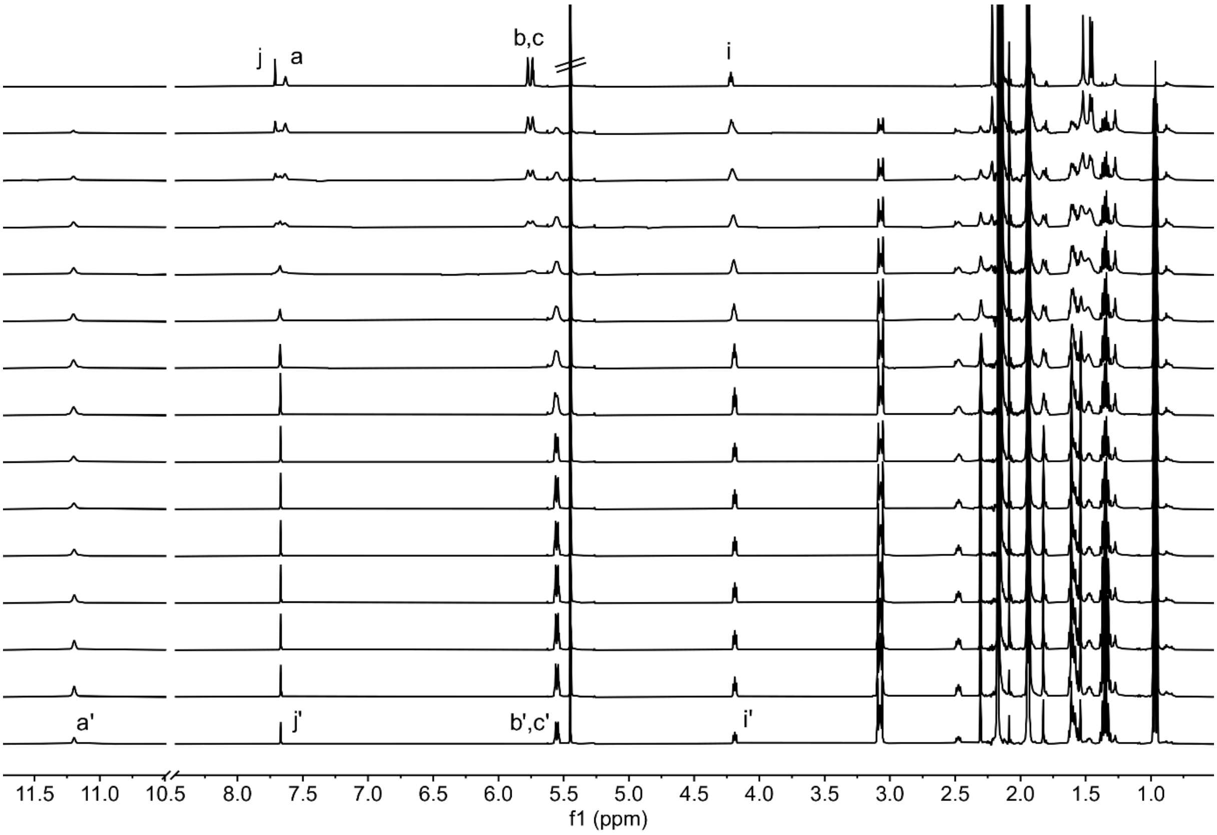

Figure S15. ^{1}H NMR spectral changes (500 MHz, 293 K) of (*o*)-**1** in MeCN-*d*$_3$ (1.83 mM) upon the stepwise addition of NBu_4Cl (from top to bottom: 0.00, 0.23, 0.34, 0.44, 0.54, 0.64, 0.73, 0.82, 1.00, 1.16, 1.38, 1.71, 2.24, 2.65, 2.83 equivalents).

Titration of NBu_4Br to (*o*)-1:

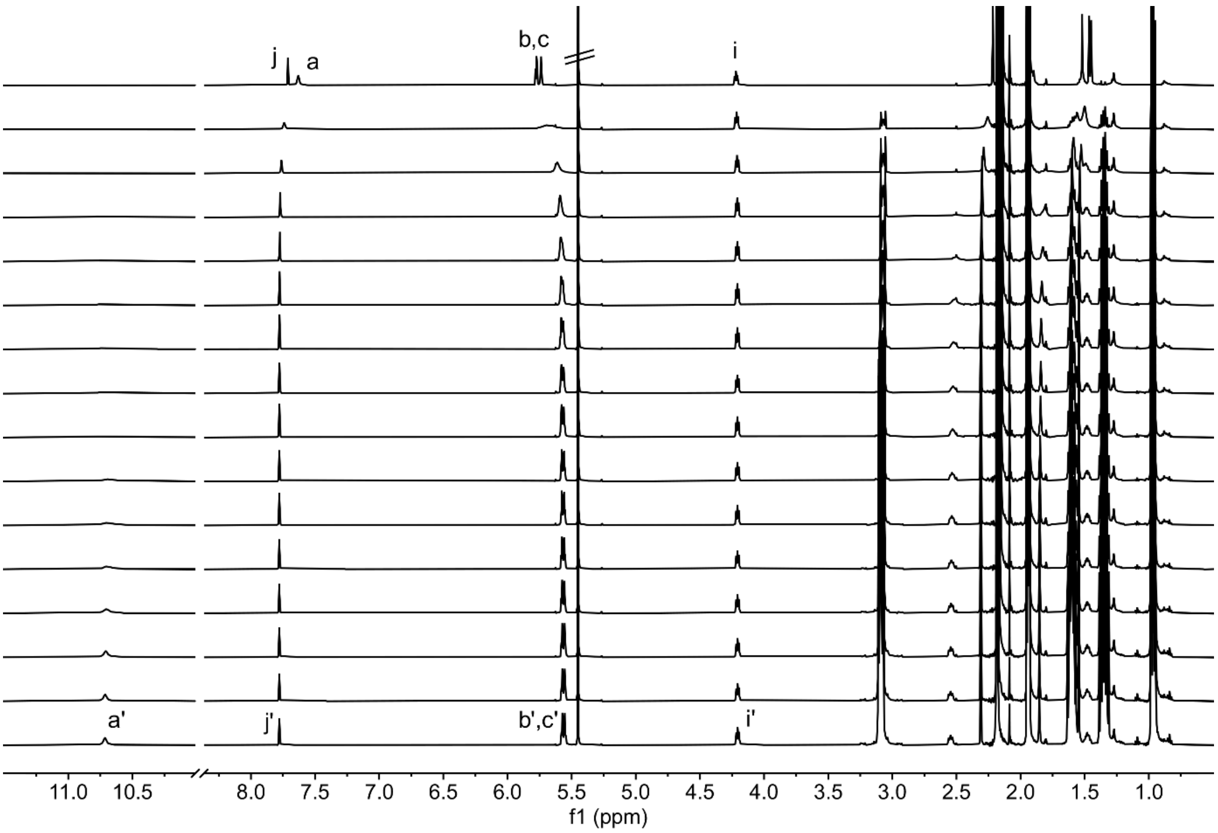

Figure S16. ^{1}H NMR spectral changes (500 MHz, 293 K) of (*o*)-**1** in MeCN-d_3 (1.83 mM) upon the stepwise addition of NBu_4Br (from top to bottom: 0.00, 0.38, 0.75, 1.10, 1.44, 1.77, 2.09, 2.39, 2.69, 3.25, 3.77, 4.50, 5.57, 7.31, 8.66, 9.33 equivalents).

Data fitting for the titration of NBu_4Br to (*o*)-1:

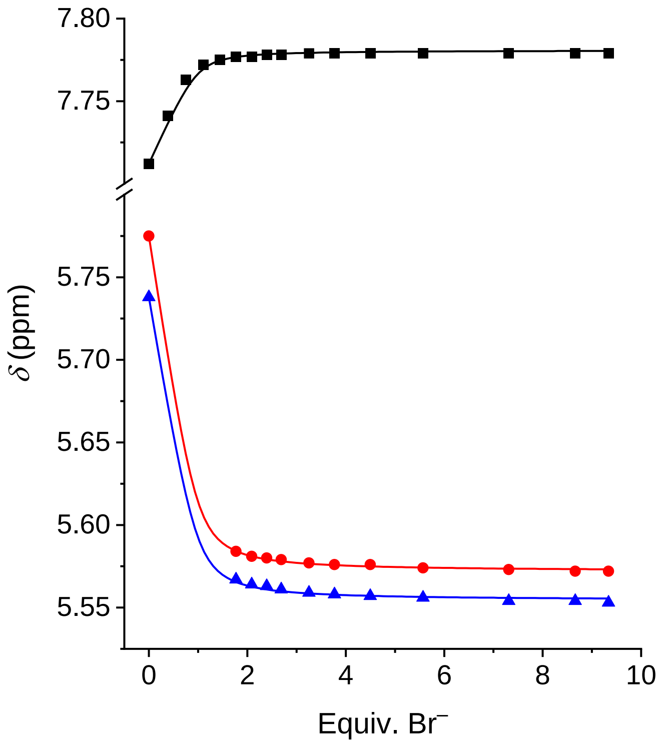

Figure S17. Titration curves for the addition of NBu_4Br to (*o*)-**1** in MeCN-d_3 and data fitting to a 1:1 binding model using HypNMR[4] by simultaneous analysis of thienyl (H_j; black) and β-pyrrolic ($H_{b,c}$; red and blue) ^1H NMR signals: $K_a = 1.02 \times 10^4$ M^{-1}.

Titration of NBu4I to (*o*)-1:

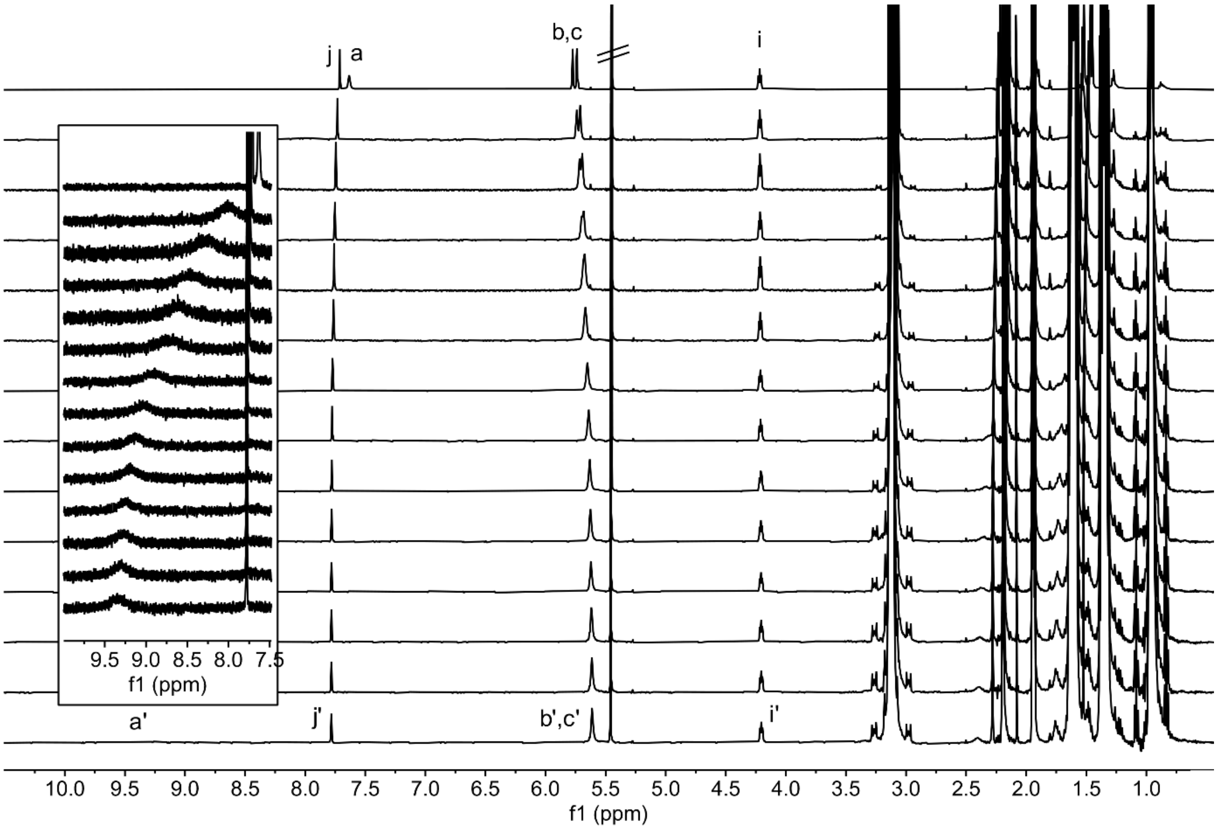

Figure S18. ^{1}H NMR spectral changes (500 MHz, 293 K) of (*o*)-**1** in MeCN-d_3 (1.83 mM) upon the stepwise addition of NBu4I (from top to bottom: 0.00, 4.83, 9.31, 13.5, 17.3, 21.0, 29.0, 35.9, 41.9, 47.2, 51.8, 55.9, 59.6, 62.9 equivalents).

Data fitting for the titration of NBu_4I to (*o*)-1:

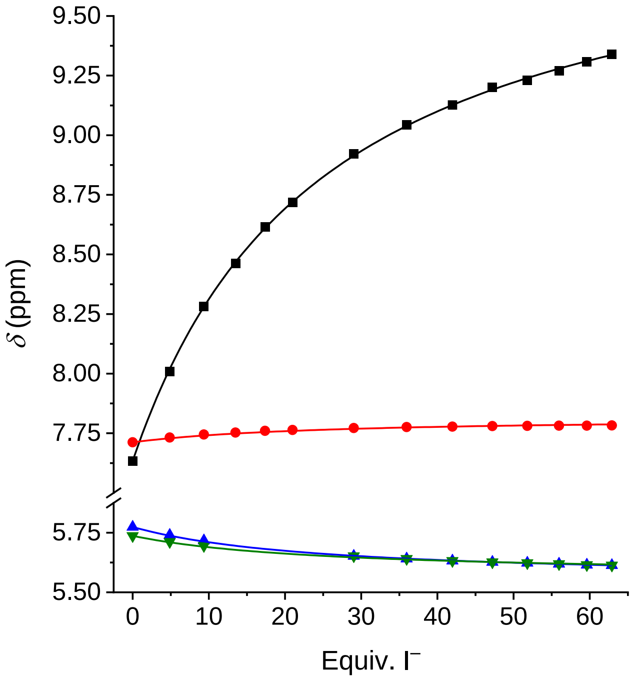

Figure S19. Titration curves for the addition of NBu_4I to (*o*)-**1** in MeCN-d_3 and data fitting to a 1:1 binding model using HypNMR[4] by simultaneous analysis pyrrole-NH (H_a; black), thienyl (Hj; red) and β-pyrrolic ($H_{b,c}$; blue, green) ^{1}H NMR signals: $K_a = 23\ M^{-1}$.

Titration of NBu_4Cl to (*o*)-1/(*c*)-1:

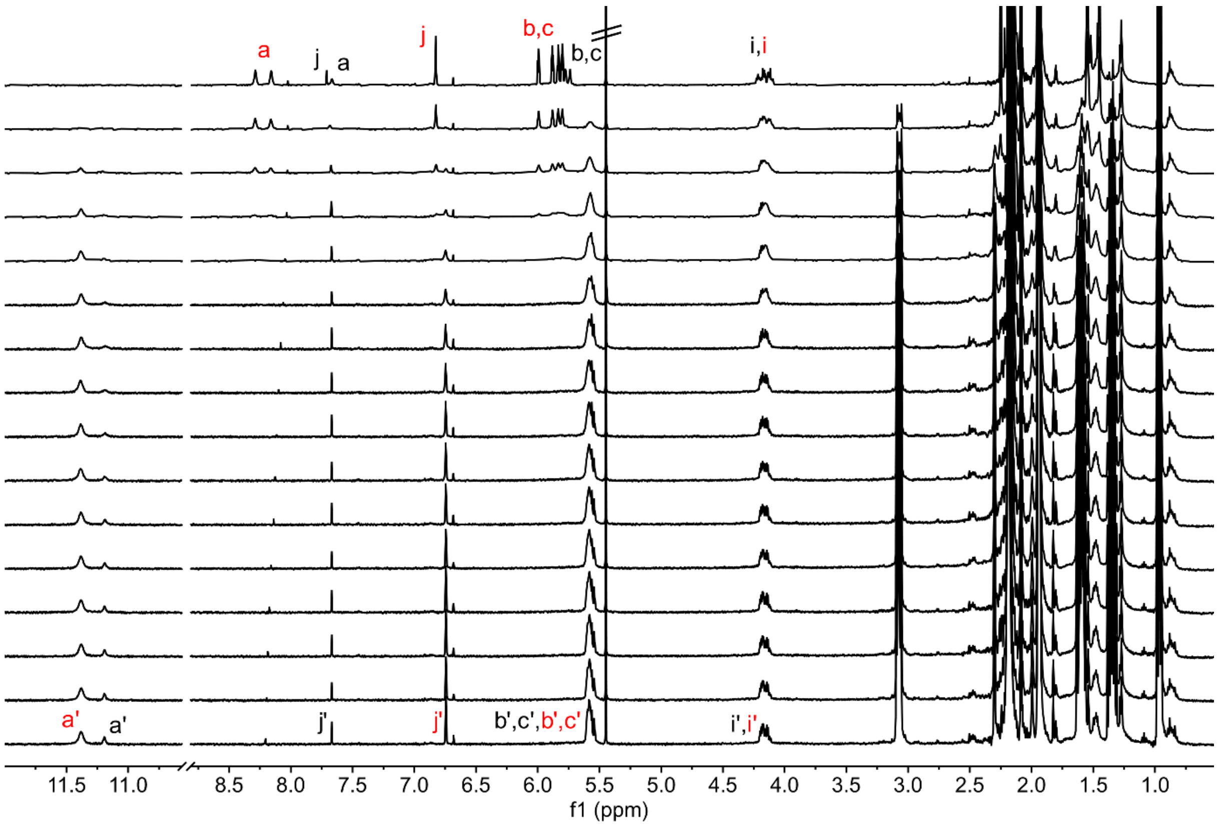

Figure S20. ^{1}H NMR spectral changes (500 MHz, 293 K) of a 81:19 (*c*)-**1**/(*o*)-**1** mixture in MeCN-d_3 (2.32 mM) upon the stepwise addition of NBu_4Cl (from top to bottom: 0.00, 0.16, 0.31, 0.46, 0.60, 0.73, 0.86, 0.99, 1.11, 1.23, 1.35, 1.56, 1.77, 1.96, 2.14, 2.31 equivalents).

The binding constant ratio was determined by integration of the pyrrole-NH (H_a, $H_{a'}$) signals using the first three ^{1}H NMR spectra. The ratio was calculated using the following equation:

$$\frac{K_a(c)}{K_a(o)} = \frac{[(o)-\mathbf{1}]\,[(c)-\mathbf{1}\subset Cl^-]}{[(c)-\mathbf{1}]\,[(o)-\mathbf{1}\subset Cl^-]}$$

The integration ratios between chloride-bound ring-closed and -open isomers were 60:40, 73:27, and 77:23 in the presence of 0.16, 0.31, and 0.46 equivalents, respectively. These ratios were used to estimate the concentrations of each species, affording $K_a(c)/K_a(o) = 0.46 \pm 0.16$.

Titration of NBu_4Br to (*o*)-1/(*c*)-1:

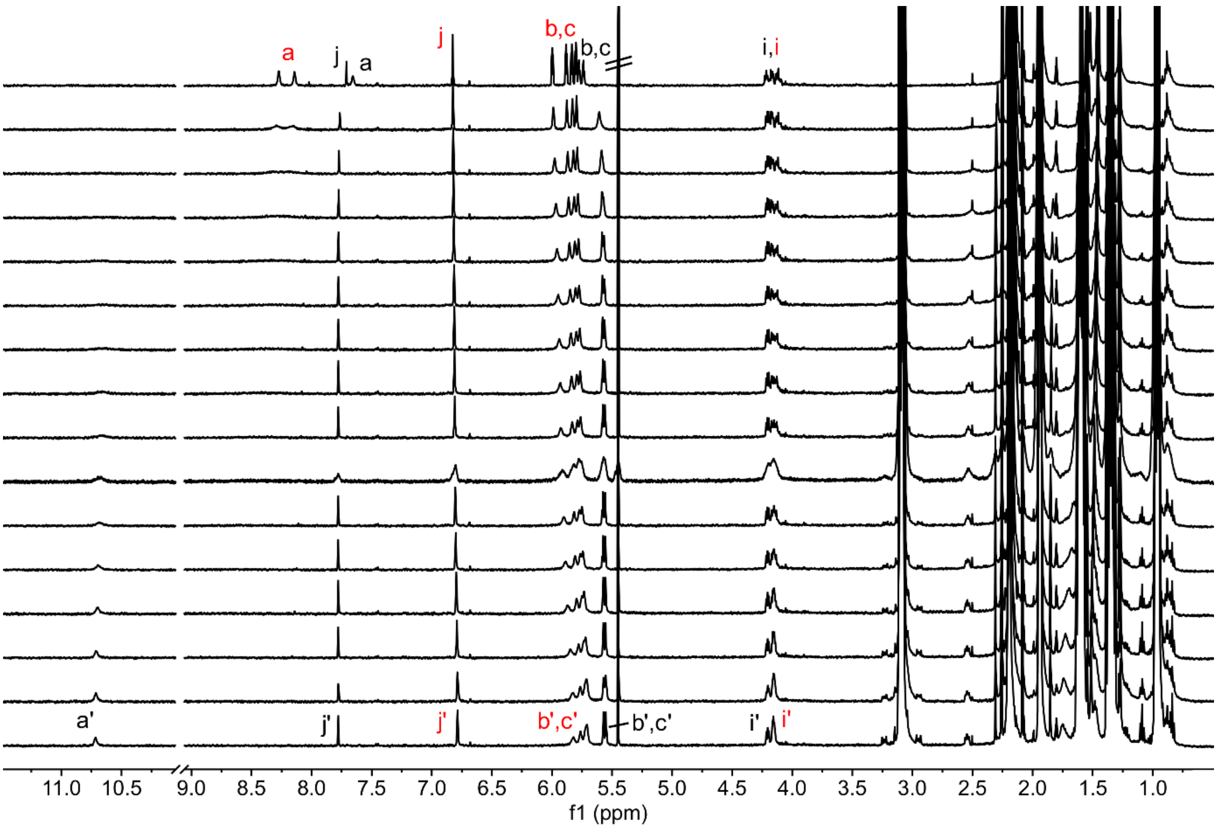

Figure S21. ^{1}H NMR spectral changes (500 MHz, 293 K) of a 73:27 (*c*)-**1**/(*o*)-**1** mixture in MeCN-d_3 (2.54 mM) upon the stepwise addition of NBu_4Br (from top to bottom: 0.00, 0.34, 0.66, 0.97, 1.27, 1.56, 1.83, 2.10, 2.36, 2.85, 3.31, 3.95, 4.89, 6.42, 7.61, 8.29 equivalents).

Data fitting for the titration of NBu_4Br to (*o*)-1/(*c*)-1:

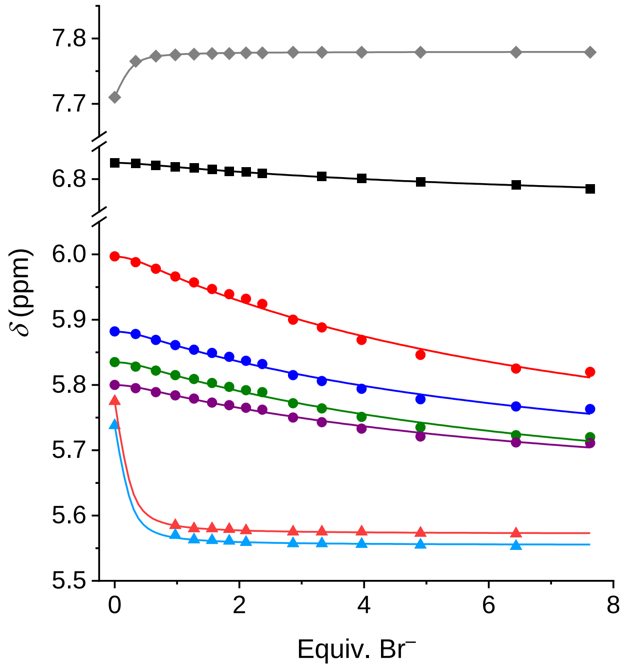

Figure S22. Titration curves for the addition of NBu_4Br to a 73:27 (*c*)-**1**/(*o*)-**1** mixture in MeCN-d_3 and data fitting considering 1:1 binding to each isomer using HypNMR,[4] by simultaneous analysis of thienyl [H_j; black for (*c*)-**1** and grey for (*o*)-**1**] and β-pyrrolic [$H_{b,c}$; red, blue, green, purple for (*c*)-**1** and light red, light blue for (*o*)-**1**] ^{1}H NMR signals, where the stability constant of (*o*)-**1**⊂Br^- was set as constant [$K_a(o) = 8.77 \times 10^3$ M^{-1}, as was determined by ITC titrations] and the stability constant of (*c*)-**1**⊂Br^- was refined: $K_a(c) = 54$ M^{-1}.

Titration of NBu_4I to (*o*)-1/(*c*)-1:

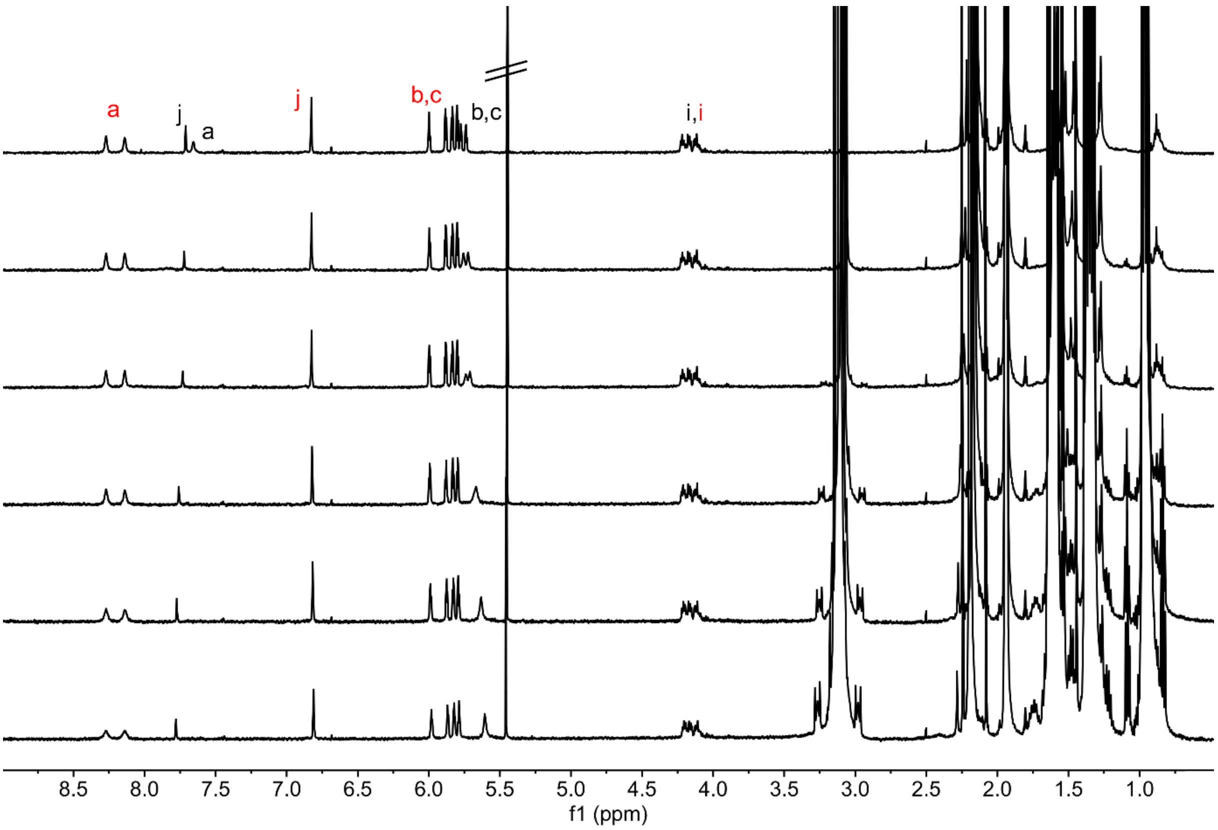

Figure S23. ^{1}H NMR spectral changes (500 MHz, 293 K) of a 72:28 (*c*)-**1**/(*o*)-**1** mixture in MeCN-d_3 (2.54 mM) upon the stepwise addition of NBu_4I (from top to bottom: 0.00, 1.79, 3.51, 12.6, 26.2, 45.7 equivalents); $K_a < 1$ M^{-1} (taking into account <10% association in the presence of 45.7 equivalents, where K_a = [(*c*)-**1**$\subset$I$^-$]/[(*c*)-**1**][I$^-$].

ITC titration experiments

The titrations were carried out by adding 6 μL aliquots of a solution of (*o*)-**1** in degassed MeCN to an approximately 17 times more concentrated solution of tetrabutylammonium chloride or tetrabutylammonium bromide in the same solvent at 298 K. To calculate the association constants and thermodynamic parameters, the data (measured in duplo) was fitted to a "one set of sites" binding model using the Origin software in the ITC module.

Titration of NBu_4Cl with (*o*)-1:

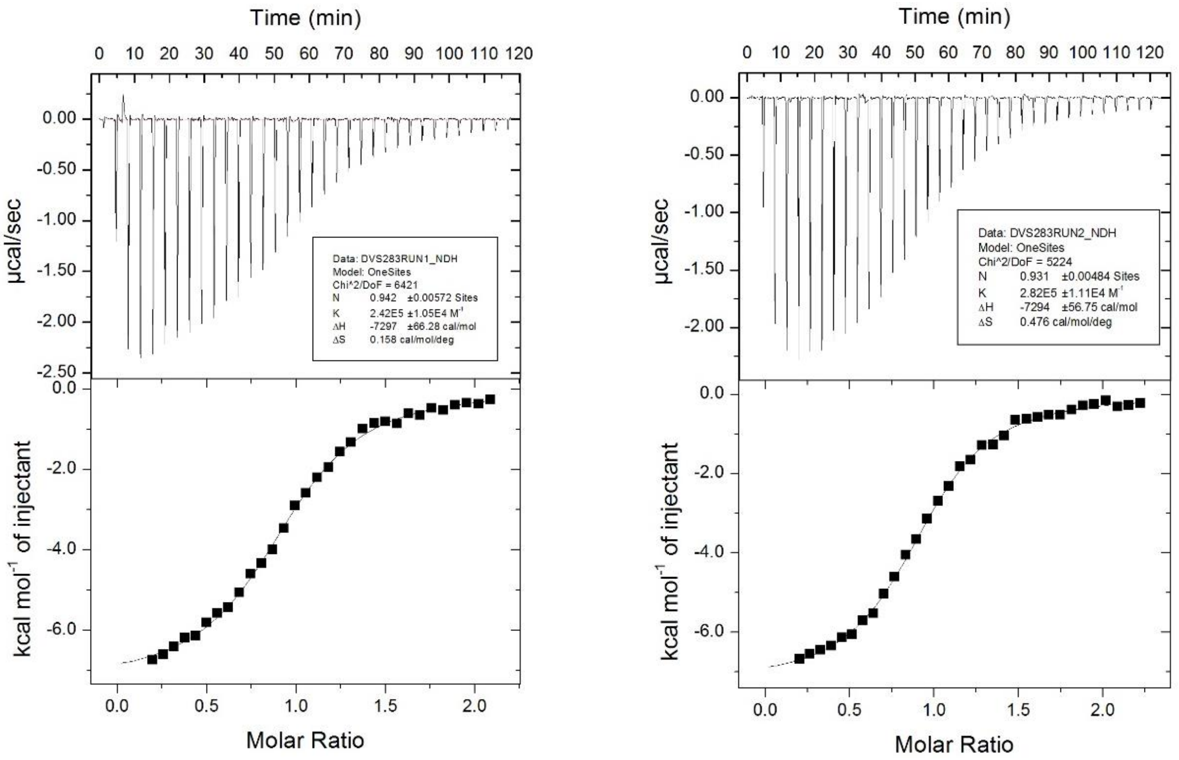

Figure S24. Binding isotherms for the calorimetric titration of (*o*)-**1** (1.06 mM) to NBu_4Cl (0.063 mM) in MeCN at 298 K and data fitting (continuous line) to a 1:1 binding model: K_a = 2.62 ± 0.20 × 10^5 M^{-1}; ΔH = –7.296 ± 0.002 kcal mol^{-1}; ΔS = 0.317 ± 0.159 cal mol^{-1}.

Titration of NBu_4Br with (*o*)-1:

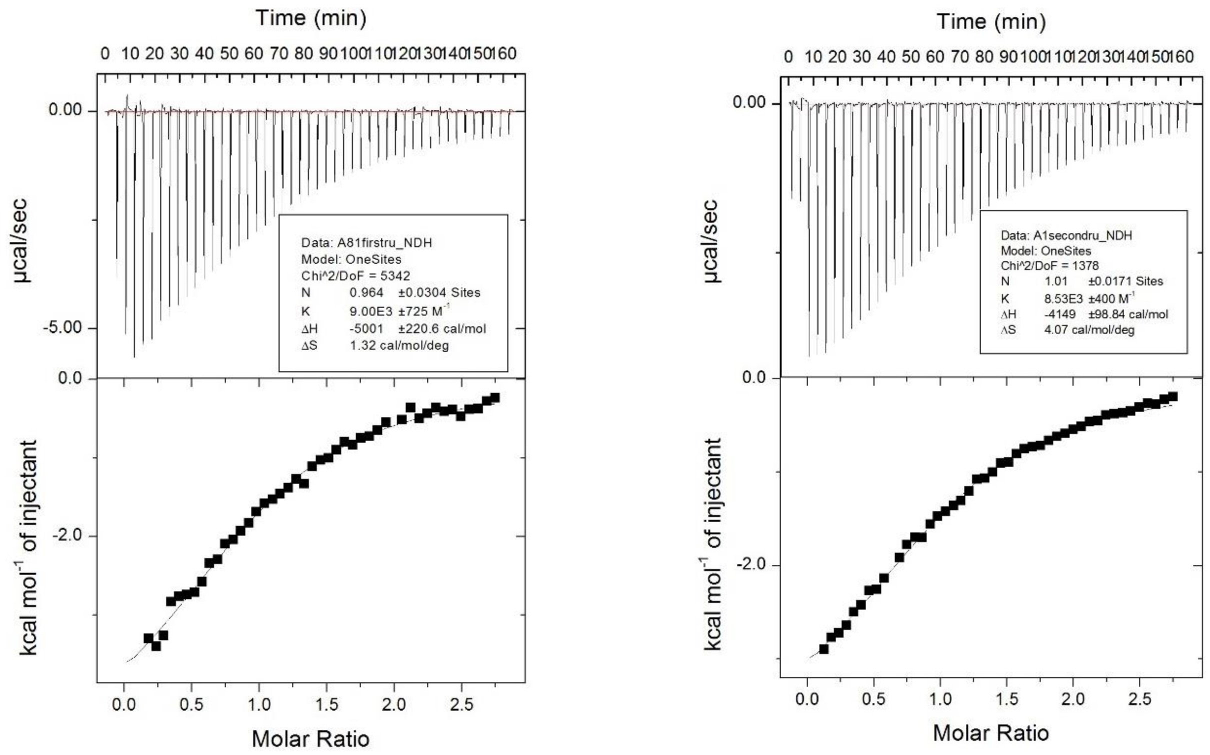

Figure S25. Binding isotherms for the calorimetric titration of (*o*)-**1** (4.97 mM) to NBu_4Br (0.297 mM) in MeCN at 298 K and data fitting (continuous line) to a 1:1 binding model: K_a = 8.77 ± 0.24 × 10^3 M^{-1}; ΔH = –4.575 ± 0.426 kcal mol^{-1}; ΔS = 2.69 ± 1.38 cal mol^{-1}.

References

1. S. Hermes, G. Dassa, G. Toso, A. Bianco, C. Bertarelli and G. Zerbi, *Tetrahedron Lett.* 2009, **50**, 1614–1617.
2. (*a*) D.-W. Yoon, H. Hwang and C.-H. Lee, *Angew. Chem. Int. Ed.* 2002, **41**, 1757–1759; (*b*) D.-W. Yoon, D. E. Gross, V. M. Lynch, J. L. Sessler, B. P. Hay and C.-H. Lee, *Angew. Chemie Int. Ed.* 2008, **47**, 5038–5042.
3. G. M. Sheldrick. *Acta Cryst*, 2015, **C71**, 3–8.
4. C. Frassineti, S. Ghelli, P. Gans, A. Sabatini, M. S. Moruzzi and A. C. Vacca, *Anal. Biochem.* 1995, **231**, 374–382.
